# Comparative Aerial and Ground Based High Throughput Phenotyping for the Genetic Dissection of NDVI as a Proxy for Drought Adaptive Traits in Durum Wheat

**DOI:** 10.3389/fpls.2018.00893

**Published:** 2018-06-26

**Authors:** Giuseppe E. Condorelli, Marco Maccaferri, Maria Newcomb, Pedro Andrade-Sanchez, Jeffrey W. White, Andrew N. French, Giuseppe Sciara, Rick Ward, Roberto Tuberosa

**Affiliations:** ^1^Department of Agricultural Sciences, University of Bologna, Bologna, Italy; ^2^Maricopa Agricultural Center, University of Arizona, Tucson, AZ, United States; ^3^US Arid Land Agricultural Research Center, USDA-ARS, Maricopa, AZ, United States

**Keywords:** *Triticum turgidum* L. subsp. *durum*, durum wheat, drought, high-throughput phenotyping, UAV, NDVI, GWAS, QTL

## Abstract

High-throughput phenotyping platforms (HTPPs) provide novel opportunities to more effectively dissect the genetic basis of drought-adaptive traits. This genome-wide association study (GWAS) compares the results obtained with two Unmanned Aerial Vehicles (UAVs) and a ground-based platform used to measure Normalized Difference Vegetation Index (NDVI) in a panel of 248 elite durum wheat (*Triticum turgidum* L. ssp*. durum* Desf.) accessions at different growth stages and water regimes. Our results suggest increased ability of aerial over ground-based platforms to detect quantitative trait loci (QTL) for NDVI, particularly under terminal drought stress, with 22 and 16 single QTLs detected, respectively, and accounting for 89.6 vs. 64.7% phenotypic variance based on multiple QTL models. Additionally, the durum panel was investigated for leaf chlorophyll content (SPAD), leaf rolling and dry biomass under terminal drought stress. In total, 46 significant QTLs affected NDVI across platforms, 22 of which showed concomitant effects on leaf greenness, 2 on leaf rolling and 10 on biomass. Among 9 QTL hotspots on chromosomes 1A, 1B, 2B, 4B, 5B, 6B, and 7B that influenced NDVI and other drought-adaptive traits, 8 showed *per se* effects unrelated to phenology.

## Introduction

Global warming and the increasing frequency and severity of drought events unequivocally underline the urgency to select crops able to sustain growth in rainfed conditions, particularly when grown in Mediterranean countries, where climatic change is expected to exacerbate yield uncertainty (Ortiz et al., 2008; Kelley et al., 2015; Kyratzis et al., 2017). The selection of drought-resistant cultivars increasingly relies on the use of yield-related proxies selected either directly (Reynolds and Tuberosa, 2008) or via marker-assisted selection once the quantitative trait loci (QTLs) underpinning the variability of the relevant trait are identified (Langridge and Reynolds, 2015; Maccaferri et al., 2016; Mason et al., 2018).

The recent progress in high-throughput phenotyping platforms (HTTPs) based primarily on the use of ground-based and/or Unmanned Aerial Vehicles (UAVs) provides unprecedented opportunities to accurately measure proxy traits in hundreds of plots (Pauli et al., 2016; Duan et al., 2017; Shakoor et al., 2017; Shi et al., 2017; Trapp et al., 2017), as required in experiments to identify QTLs. In this respect, increasing attention is being devoted to the use of ground-based and aerial HTPPs that allow for such high-throughput phenotyping levels (Araus and Cairns, 2014; Zaman-Allah et al., 2015; Kefauver et al., 2017; Madec et al., 2017). A potential limitation of ground-based phenotyping platforms is the considerably longer time required to complete the measurements as compared to UAV-based remote sensing which allows phenotyping over a larger area in less time, an important prerequisite to minimize the effects due to daily fluctuations in environmental conditions, inevitable in large-scale experiments (Tuberosa, 2012). However, a potential advantage of ground-based platforms is the increased data resolution as result of shorter distances between sensors and plant targets. Empirical data are needed to compare benefits of the different platforms for different experimental objectives.

Because water shortage affects vegetative state and cover, drought-stress monitoring can be based on the use of vegetation indices (VIs). Normalized Difference Vegetation Index (NDVI) was found to be an effective indicator of vegetation response to drought based on the relationships between NDVI and a meteorologically based drought index (Ji and Peters, 2003). NDVI is based on the difference between the maximum absorption of radiation in the Red spectral region (from 620 to 690 nm) as result of chlorophyll pigments and the maximum reflectance in near infrared (NIR, from 760 to 900 nm) light as result of the leaf cellular structure (Tucker, 1979). Healthy and living canopies absorb most of the Red light by the photosynthetic pigments, while the NIR light is mostly reflected due to light scattering in leaf internal structure and canopy architecture. Therefore, NDVI-value, computed as (NIR – Red)/(NIR + Red), integrates biomass (or leaf area) and leaf chlorophyll content (Lukina et al., 1999), hence providing a proxy for grain yield (Labus et al., 2010). In wheat, NDVI has been shown to be associated with drought-adaptive traits as well as grain yield under stressed conditions (Bort et al., 2005; Marti et al., 2007; Reynolds et al., 2007; Lobos et al., 2014; Bowman et al., 2015; Tattaris et al., 2016; Yousfi et al., 2016), which ultimately allows for the identification of the relevant QTL governing the adaptive response to drought. In this case, it is important to account for the effects of the single QTLs on flowering time, a trait well known to influence drought adaptation (Tuberosa, 2012). A number of key genes (*PPD-A1, PPD*-*B1, FT-7A*-indel, *Rht-B1b*, and *VRN-A1*) affect flowering time and, consequently, NDVI and other drought-adaptive traits (Milner et al., 2016). Therefore, their effects should be accounted for when interpreting the results of QTL analyses, particularly when aiming at identifying loci that affect drought resistance on a *per se* basis, i.e., irrespectively of indirect effects due to differences in flowering time.

Although remote sensing based on the utilization of UAVs equipped with either conventional or hyperspectral and multispectral cameras is being increasingly adopted as an alternative to portable cameras and spectroradiometers to measure NDVI in wheat (Haghighattalab et al., 2016; Holman et al., 2016; Yang et al., 2016; Kyratzis et al., 2017) no study has yet compared the QTL results of a genome-wide association study (GWAS) for NDVI measured with both aerial- and ground-based phenotyping platforms in crops under both well-watered and water-deficit conditions of increasing severity. To our best knowledge, this study is the first to report on the use of UAV-based NDVI remote sensing for GWAS analysis in crops and to compare the results with those obtained via a ground-based HTPP. Importantly, GWAS of NDVI and other drought-adaptive traits allowed us to identify a number of QTL hotspots with *per se* effects that provide suitable targets for enhancing drought tolerance via marker-assisted selection.

## Materials and methods

### Plant material and field management

The field trial was conducted at Maricopa Agricultural Center (33.070° N, 111.974° W, elevation 360 m) on a Casa Grande soil (fine-loamy, mixed, superactive, hyperthermic Typic Natrargids) (Supplementary Figure 1). The plant material included 248 accessions of durum wheat from the association mapping population UNIBO-Durum Panel (hereafter referred to as “Durum Panel”) assembled at the University of Bologna (UNIBO), representing a large portion of the genetic diversity present in the most important improved durum wheat gene pools.

The Durum Panel includes Mediterranean-adapted accessions selected and released from breeding programs in Italy, the International Maize and Wheat Improvement Center (CIMMYT), the International Center for Agricultural Research in the Dry Areas (ICARDA), the National Institute for Agricultural Research (INRA, France) and the Institute of Agrifood Research and Technology (IRTA, Spain). The Durum Panel also includes accessions released by public breeding programs in the Northern Great Plains of the USA and Canada (North Dakota, Montana, Saskatchewan and Alberta), private French breeders and Australian breeding programs, as well as representative accessions from the Pacific Southwest of the US, commonly referred to as “Desert-Durum®” (Supplementary Table 1).

The 248 accessions were planted on 20 December 2016 according to a Randomized Complete Block Design (RCBD) with two replicates and border plots (cv. Orita). Each accession was evaluated in two-row plots (3.5 m long, 0.76 m apart) with a final density of 22 plants/m^2^. Before planting, nitrogen at 112 kg ha^−1^ and phosphorus (P_2_0_5_) at 56 kg ha^−1^ were incorporated into the soil and 28 days after sowing, irrigation was managed by a pressurized drip system using lines buried ~10 cm deep. Drip irrigation was stopped on 16 March 2017 and from that date the accessions were subjected to a progressive drought stress until 3–4 April 2017 when plants were harvested to measure biomass.

Soil moisture data were collected for monitoring the water stress conditions using time domain reflectometry (TDR) probes (rod length: 15 cm) on 22 and 23 March 2017. TDR probes worked in 8 equidistant field ranges by inserting the rods into the soil and within a few seconds the moisture value is presented on a display unit.

Plants were harvested on 105 days after planting (DAP) to allow for planting the next phenotyping experiment and therefore biomass data indicate the status at a point in time rather than direct estimates of final yields.

Disease and insect pest pressure were negligible throughout the crop.

### Leaf water status

Relative Water Content (RWC) was measured in flag leaves collected from the two replicates for the cultivars “Gallareta,” “Karim,” “Mexicali 75,” and “Svevo.” Flag leaves were sampled on 24 March 2017 (DAP: 94), 27 March 2017 (DAP: 97) and 31 March 2017 (DAP: 101) placed in glass containers within a cooler and transported immediately to the laboratory to minimize water loss due to evaporation. Samples were weighed as fresh weight (FW) and then submerged in distilled water. After rehydration for 24 h at 4°C in the dark, the turgid leaves were rapidly blotted to remove surface water and weighed to obtain turgid weight (TW). Finally, the leaves were oven-dried at 60°C for 24 h and then the dry weight (DW) was obtained. RWC-values were computed as follows: [(FW – DW)/(TW – DW)] × 100 (Barrs, 1968).

### NDVI measurements

NDVI was measured on progressive days after planting using two UAV-based and one tractor-based platforms and related phenology of each accession was evaluated on the basis of the Zadoks scale (Supplementary Table 2).

UAV-based NDVI was extracted from georeferenced orthomosaic GeoTIFFs generated from imagery captured from autopiloted flights of either a MicaSense RedEdge multi-spectral camera (MicaSense, Seattle, WA) carried on a hexacopter, or a Parrot Sequoia (Parrot, Paris, France) multi-spectral camera carried on an eBee (SenseFly, Lausanne) fixed wing aircraft. Table 1 compares features of the two multispectral cameras in terms of band centers and bandwidths.

**Table 1:** Properties of Sequoia, RedEdge, and GreenSeeker Normalized Difference Vegetation Index (NDVI) sensors and including type of recorded spectral band, bandcenter, and bandwidth.

**Sensor**	**Spectral band**	**Band center (nm)**	**Band width (nm)**
UAV-Sequoia^a^	Green	550	40
	Red	660	40
	Red Edge	735	10
	NIR	790	40
	Blue	475	20
Tractor-GreenSeeker^b^	Red	660	25
	NIR	770	25
UAV-RedEdge^c^	Green	560	20
	Red	668	10
	Red Edge	717	10
	NIR	840	40

a https://www.micasense.com/parrotsequoia/

b https://agriculture.trimble.com/precision-ag/products/greenseeker/

c https://www.micasense.com/

Flights were conducted at 40–42 m above ground level, resulting in ground sampling distances of ~3 cm/pixel for the RedEdge, and 4.4 cm/pixel for the Sequoia. Mission planning was done with UgCS (UgCS, Riga) for the RedEdge camera, and either eMotion 3 (senseFly, Lausanne) or Atlas Flight (MicaSense, Seattle, WA) for the Sequoia camera. All flights were planned for 80% image overlap along flight corridors. Both the Sequoia and RedEdge cameras use global shutters.

Pix4DMapperPro desktop software (Pix4D SA, Switzerland, http://pix4d.com) was used to generate orthomosaics for each camera band. Six to eight ground control points (GCP) geolocated with Real Time Kinematic (RTK) survey precision were used to georeference the orthomosaics. Camera images were calibrated using manufactured supplied reflectance panels that were imaged at the beginning of each flight. The Pix4D processing options were essentially the same as those of Pix4D's “Ag Multispectral” template version 4.1.10, except that GeoTIFF tiles were merged to create the NDVI orthomosaic.

Plot-level NDVI means from UAV's were created in QGIS software version 2.18.3 (QGIS, US, http://www.qgis.org). Shape files containing annotated single plot polygons were generated with an R (r-project.org) script. Shape files with GCPs as features (points) were also employed based on RTK survey grade measuring devices. For all flights, the GeoTIFF with the NDVI orthomosaic from Pix4D was combined with the plot polygon and GCP shape files in a single QGIS project. Confirmation of proper geolocations of the Pix4D orthomosaics was achieved by visually confirming alignment of the visible GCPs with the corresponding points in the feature shape file. NDVI plot means were generated using the Zonal Statistics function in QGIS.

The tractor-based system was similar to that described by Andrade-Sanchez et al. (2013) but carried five GreenSeeker spectral sensors and RT200 communication module (Trimble, Inc., Sunnyvale, CA) mounted in a frame at the front of the vehicle. These active sensors are equipped with their own source of modulated white light, which is directed toward the top of the crop canopy with the platform in motion at an average speed of 0.84 m s^−1^. A portion of the sensor-generated light reflects off the crop and is measured by Red and Near Infrared (NIR) wide-band filters located in the sensor head. The height position of the sensors was set to 1.32 m above ground in every event. Since the approximate view angle of this sensor model is 28°, the field-of-view (FOV) of each sensor was ~50-cm at the soil surface. The ground platform was retrofitted with an ultra-precise RTK Global Navigation Satellite System (GNSS) receiver, AgGPS332 (Trimble, Inc., Sunnyvale, CA) to generate positioning data via “GGA” National Marine Electronics Association (NMEA) messages. The data acquisition system used in the tractor platform was a CR3000 micro-logger (Campbell Scientific, Logan, UT) programmed to record the NDVI output of all five spectral sensors plus latitude and longitude coordinates at a rate of 5 Hz. The combination of data sampling frequency and platform speed of operation produced an average of 20 NDVI data points for each plot.

The lme4 package (r-project) and custom R scripts were used to conduct a spatial adjustment analysis of the raw NDVI plot data from aerial- and ground-based platforms using a mixed procedure including row and column random effects and a moving mean of variable size for optimizing spatial adjustment. Repeatability values and Pearson's correlation *r* coefficients among growth stages were also calculated in R.

### Phenology score (Zadoks system), leaf chlorophyll content (SPAD), leaf rolling, and dry biomass evaluation

Phenology of each accession was evaluated on the basis of the Zadoks scale (Zadoks et al., 1974) (Supplementary Table 2).

Flag leaf “greenness” on 101 DAP was assessed based on Soil-Plant Analysis Development (SPAD) estimates obtained with a non-destructive chlorophyll meter SPAD-502Plus (Konica Minolta Sensing, Inc., Japan) as an indicator of leaf photosynthetic activity, chlorophyll content and nitrogen (N) status. The hand-held SPAD meter operates by an illuminating system that emits Red (650 nm) and infrared (940 nm) light transmitted through a leaf to a receptor.

Leaf rolling (LR) was visually estimated on 99 DAP with a score from 0 (no leaf rolling) to 9 (severely rolled).

At the end of the field trial, plants within the entire two-row plots were cut with mechanical harvester (Carter mfg equipment) while subsamples of 2–3 plants were collected to evaluate moisture content in order to estimate dry biomass on 3–4 April 2017. Dry weight of the harvested plot assumed plot dimensions of 1.5 m width and 3.5 m length and was adjusted to 0% moisture. Plant moisture content (%) at harvest was estimated from a subsample of biomass either placed directly in a drying oven or stored temporarily in an uncooled greenhouse that reached a diurnal high temperature of 60°C before being transferred to an oven at 60°C for final drying.

### SNP genotyping, population structure, and GWAS model

For each accession, genomic DNA was extracted using NucleoSpin® 8/96 Plant II Core Kit from Macherey Nagel and sent for SNP genotyping to TraitGenetics (http://www.traitgenetics.com/en/).

The Illumina iSelect 90K wheat SNP assay (Wang et al., 2014) was used and genotype calls were obtained as described in Maccaferri et al. (2015b). The tetraploid-consensus-2015 reported in Maccaferri et al. (2015a) was used to assign polymorphisms to chromosomes and map positions.

Linkage disequilibrium (LD) among markers was calculated in HaploView 4.2 software (Barrett et al., 2005), for each chromosome of A and B genomes and only SNPs with known position and with a minor allele frequency > 0.05 were considered. LD decay pattern as a function of consensus genetic distances was inspected considering squared allele frequency correlation (*r*^2^) estimates obtained for all pairwise comparisons among intra-chromosomal SNPs. Curve fit and distance at which LD decays below *r*^2^ 0.3 were used to define the confidence intervals of QTLs detected in this study as already reported for the same germplasm by Liu et al. (2017) using a custom script in R following the methodology described in Rexroad and Vallejo (2009) and in Maccaferri et al. (2015a).

Population structure was assessed in STRUCTURE software 2.3.4 (Pritchard et al., 2000) using a reduced subset of 2,382 markers pruned for *r*^2^ = 0.5 using the corresponding tagger function in Haploview 4.2 (Barrett et al., 2005).

The model-based quantitative assessment of subpopulation memberships of the accessions was carried out in STRUCTURE using inferences based on molecular SNP data only. STRUCTURE model included admixture and correlated allele frequencies among subpopulations. Numbers of hypothetical subpopulations ranging from *k* = 2 to 10 were assessed using 50,000 burn-in iterations followed by 100,000 recorded Markov-Chain iterations. To estimate the sampling variance (robustness) of population structure inference, five independent runs were carried out for each *k*.

The rate of change in the logarithm of the probability of likelihood [Ln*P*(D)] value between successive *k*-values (Δ*k* statistics, Evanno et al., 2005) together with the inspection of the rate of variation (decline) in number of accessions clearly attributed to subpopulations (no. of accessions with Q membership's coefficient ≥ 0.5 and ≥ 0.7) and meaningful grouping based on pedigree and accessions' passport data were used to predict the optimal number of subpopulations. Finally, to determinate the level of differentiation among subpopulations, we considered the Fixation Index (*Fst*) among all possible population pairwise combinations.

A maximum and optimal number of eight subpopulations with accession memberships consistent with the known pedigree and passport data was chosen for subsequent analysis and GWAS results interpretation based upon the integrated analysis of (i) the derivation of the variance of the maximum likelihood estimation of the model plotted vs. increasing *k* (Δ*k*, Evanno et al., 2005) and (ii) analysis of pre-existing pedigree and passport information on the accessions included in the panel which provides an estimation of parentage among accessions. A kinship matrix of genetic relationships among individual accessions of the durum panel was calculated with all non-redundant SNP markers (7,723) using the Haploview 4.2 tagger function set to *r*^2^ = 1.0. Kinship based on Identity-by-State (IBS) among accessions was calculated in TASSEL (Trait Analysis by aSSociation, Evolution and Linkage) 5.2.37.

Subsequently, 17,721 SNP markers with minor allele frequency (MAF) > 0.05, imputed with LinkImpute (LDkNNi) (Money et al., 2015) in TASSEL, were used in a GWAS of NDVI, leaf chlorophyll content, leaf rolling and phenology scores (Zadoks system) on 87 and 100 DAP. Marker-trait association (GWAS) analysis was implemented in the software package TASSEL 5.2.37 with a Mixed Linear Model (MLM; Yu et al., 2006; Bradbury et al., 2007) which included either the Kinship matrix (MLM-K) alone or STRUCTURE subpopulation membership estimates plus Kinship plus (MLM-Q+K) as random effect. Following Zhang et al. (2010), MLM was specified as follows: y = Xβ + Zu + e, where y is the phenotype value, β is the fixed effect due to marker and u is a vector of random effects not accounted for by the markers; X and Z are incidence matrices that related y to β and u while e is the unobserved vector of random residual. Based on GWAS Q-Q (quantile-quantile) plot results (Supplementary Figure 2), the MLM-K was considered as the optimal model to control the *P*-value inflation associated to population structure while the MLM-Q+K model was noticed to lead to overcorrections. Thus, all GWAS analyses were subsequently carried out based on the MLM-K model. In addition, the allelic state of loci relevant for phenology (*PPD-A1, PPD*-*B1, FT-7A*-indel, *Rht-B1b*, and *VRN-A1*) was included as covariate in MLM analysis (Yu et al., 2006; Price et al., 2010). These genes are associated with the most important agronomic traits influencing NDVI and other drought-adaptive traits. GWAS *p*-values and *R*^2^ effects were extracted and QTL selection criteria was carried-out based on standard conditions of significance: “highly significant” refers to *P* < 0.0001 and “significant” refers to *P* < 0.001. The average genetic distance at which LD decayed below *r*^2^ of 0.3, a threshold frequently adopted in GWAS (Berger et al., 2013; Maccaferri et al., 2015a; Liu et al., 2017), was used to select the QTL confidence Interval (cM) in the association analysis in this study. By setting LD *r*^2^ = 0.3, the corresponding inter-marker genetic distance was 3.0 cM as reported by Liu et al. (2017). Therefore, the confidence interval of ±3.0 cM based on map positions of QTL tag-SNPs was chosen. The proportion of variance for phenotypic traits explained by selected SNPs was calculated with Minitab^1^® 18.

## Results

### Population structure and LD decay of the elite durum panel

**Figure 1:**
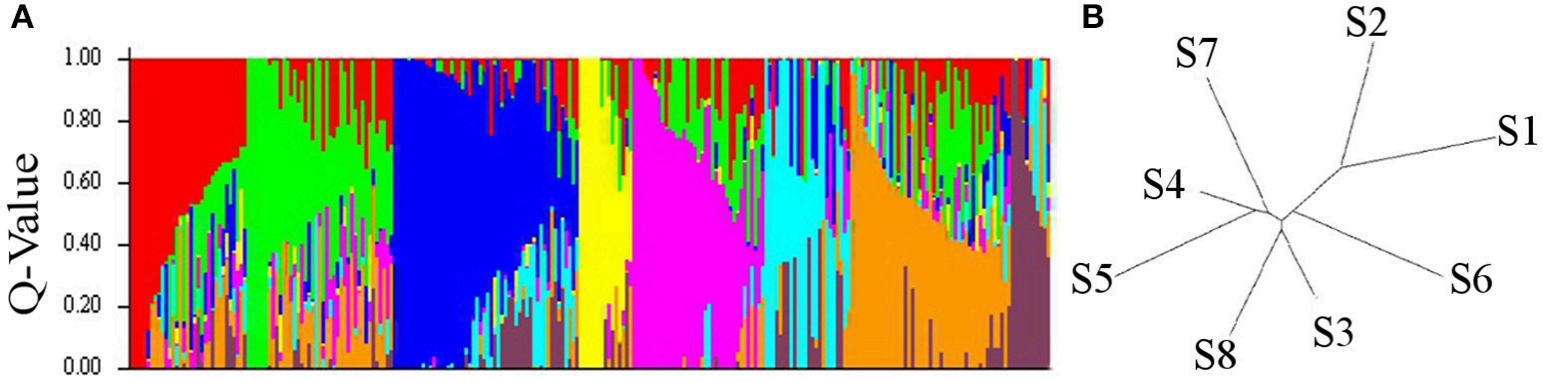
Bar Plot **(A)** and Neighbor Joining Tree **(B)** using STRUCTURE 2.3.4. for the eight durum wheat subpopulations (S1-S8) sorted by Q and relative genetic distance.

Out of the 17,721 polymorphic SNPs (minimum allele frequency ≥ 0.05) suitable for GWAS analysis, a representative reduced set of 2,382 SNPs obtained after pruning for LD at *r*^2^ = 0.50 threshold was used to investigate the population structure of the elite durum panel of 248 elite accessions. STRUCTURE analysis indicated a strong population genetic structure, as reported in previous analyses of this durum wheat germplasm, using SSR, DArT, and SNP markers (Maccaferri et al., 2011; Letta et al., 2013; Liu et al., 2017). The number of optimal *k* subpopulations ranged from five to eight. With *k* = 8, 155 accessions (62.5%) were clearly grouped into one of the eight main gene pools (Figure 1) at a *Q* membership coefficient ≥0.5, while the remaining 93 were considered as admixed.

Subgroup S1 corresponded to native Mediterranean and North African germplasm. Subgroup S2 included germplasm specifically bred for dryland areas at ICARDA (Syria) from the early 1970s. Subgroup S3 included Spanish and Moroccan cultivars from early 1970s, and CIMMYT and ICARDA selections for temperate areas. Subgroup S4 mostly included ICARDA high-yielding lines/cultivars for temperate areas and contemporary (1970s) Italian accessions obtained from cv. Creso, an important Italian founder also related to CIMMYT materials. Subgroup S5 included accessions derived from widely adapted (photoperiod insensitive) CIMMYT germplasm released in the late 1970s to early 1980s. Subgroup S6 included accessions from the mid-1970s breeding program in Italy (Valnova group) while subgroup S7 included accessions from the high-yielding CIMMYT germplasm released in the late 1980s to early 1990s (founders Altar84 and Gallareta).Finally, subgroup S8 included 40 accessions from North Dakota (USA), Canada, France and Australia (Supplementary Table 3).

The division into eight subpopulations was supported by pairwise comparisons among and within subgroups based on the Fixation Index (*Fst*) which provides a measure of subpopulation diversity (Supplementary Table 4) and by Neighbor Joining tree (Saitou and Nei, 1987; Figure 1). High genetic diversity was detected between the old Italian cultivars (S1) and the French, North American, Canadian and Australian cultivars (S8), while a considerable admixture among subgroups characterized the ICARDA, CIMMYT, and Italian groups. As a further note, only a relatively small portion of the molecular variation was accounted for by the origin of the accessions, as expected based on the high exchange rate of germplasm among breeding programs.

### Quantitative trait variation in relation to population genetic structure

**Figure 2:**
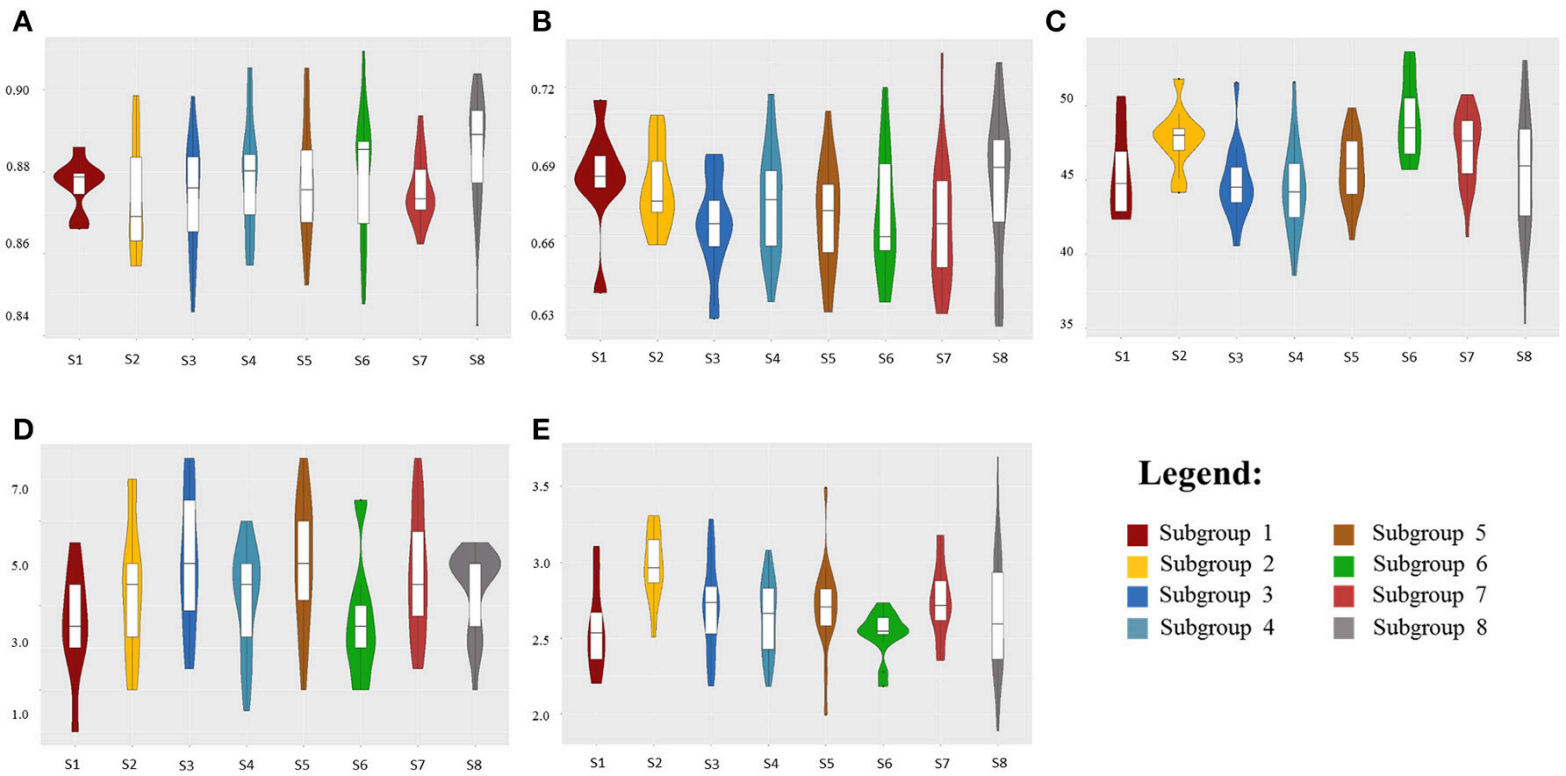
Violin-plot distributions for the eight durum wheat subpopulations (S1-S8) related to NDVI-UAV-Sequoia at 91 DAP **(A)**, NDVI-tractor-GreenSeeker at 94 DAP **(B)**, leaf chlorophyll content (SPAD) at 101 DAP **(C)**, leaf rolling at 99 DAP **(D)**, and dry biomass (ton/ha) at 105 DAP **(E)**.

Multiple linear regression was performed to estimate the impact of genetic population structure on the phenotypic traits (Supplementary Table 5). The *R*^2^-values ranged from 0.02 to 0.11 for NDVI-UAV-Sequoia scores and from 0.08 to 0.09 for NDVI-tractor-GreenSeeker scores. *R*^2^ for SPAD was higher (*R*^2^ = 0.17), reflecting the selection for high flag leaf chlorophyll content in more recent germplasm groups such as S7, while *R*^2^-values for leaf rolling and dry biomass were equal to 0.09 and 0.08, respectively. Figure 2 shows violin-plot distributions in relation to the eight subpopulations.

Although multiple regression showed a limited relationship between population structure and NDVI, violin plots and median values based on the eight subgroups evidenced trends for increased NDVI and, even more pronounced, for SPAD from the oldest subgroups (S1-S2-S3) to the most recently improved groups S5-S6-S7. Notably, subgroup S8 showed the widest within-group variation for NDVI and SPAD-values, as expected based on the concomitant presence within the same genetically highly homogeneous group of conventional plant height accessions from the Northern Plains of the US and Canada and semidwarf (*RhtB1b*) accessions from France and Austria.

### NDVI from UAV-sequoia, UAV-rededge, and ground-based greenseeker sensors

**Figure 3:**
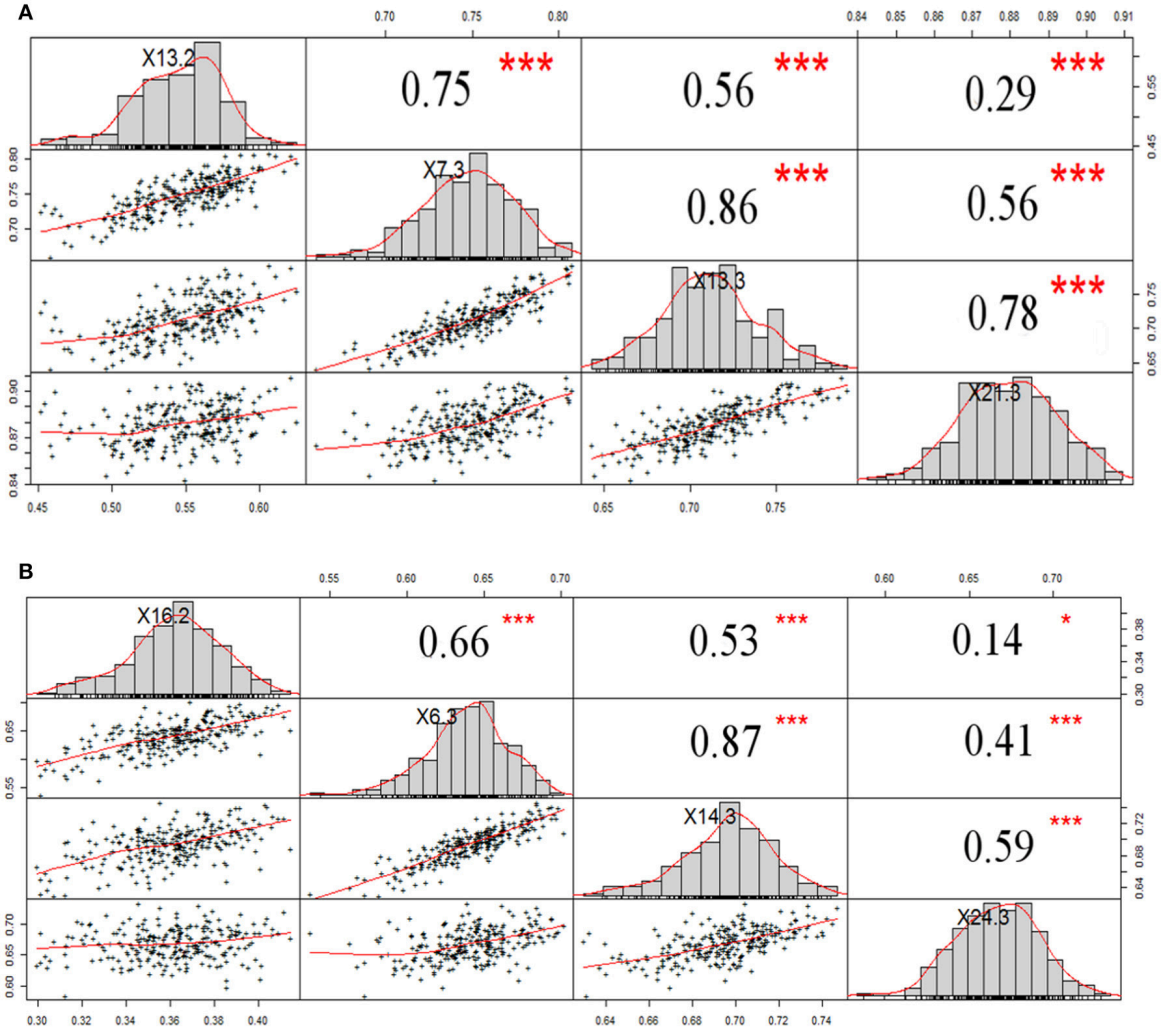
Normal distribution curves and Pearson correlation coefficients for NDVI data from tractor-GreenSeeker **(A)** and UAV-Sequoia **(B)** at four different times. ^***^P < 0.0001, ^*^0.001 < *P* < 0.01.

NDVI measurements from the aerial platforms included data from the Sequoia-sensor on four DAP associated with differing growing stages (GS), and from the RedEdge sensor on two DAP, the first of which coincided with the last measurement with the Sequoia. Phenotypic distributions approximated normality for both traits (Figure 3). Repeatability (*h*^2^) values for NDVI were mostly high for both UAV-Sequoia (from 0.77 on 55 DAP to 0.89 on 83 DAP) and UAV-RedEdge (from 0.80 on 91 DAP to 0.89 on 98 DAP) and medium-high for ground-based GreenSeeker (from 0.61 on 58 DAP to 0.67.5 on 94 DAP).

NDVI-UAV-Sequoia mean values progressively increased during the time interval from 13 February (55 DAP) (NDVI from 0.40 to 0.63) to 21 March (91 DAP) (NDVI from 0.84 to 0.91). NDVI reached the highest mean value (0.87) at 21 March (91 DAP), the last measurement. NDVI-UAV-RedEdge measurements averaged 0.82 at 21 March (91 DAP) (comparable to NDVI-UAV-Sequoia) while at 29 March (98 DAP) the mean value decreased to 0.77. Summary statistics are reported in Table 2.

**Table 2:** Summary statistics for Normalized Difference Vegetation Index (NDVI), leaf chlorophyll content (SPAD), phenology score (PHENO-score 1 and PHENO-score 2), leaf rolling (LR), and dry biomass on different days after planting (DAP) in a panel of 248 durum wheat elite advanced lines and cultivars from worldwide.

**Trait**	**DAP**	**Range**	**Mean**	**St. dev**.	***h*^*2*^(%)**
NDVI-UAV (Sequoia)	55	0.40–0.63	0.54	0.012	77.2
	77	0.66–0.81	0.74	0.029	83.9
	83	0.64–0.79	0.71	0.026	88.5
	91	0.84–0.91	0.87	0.032	87.3
NDVI-tractor (GreenSeeker)	58	0.30–0.42	0.36	0.025	61.1
	76	0.54–0.70	0.64	0.022	66.3
	84	0.63–0.75	0.69	0.028	66.9
	94	0.58–0.73	0.66	0.023	67.5
NDVI-UAV (RedEdge)	91	0.78–0.87	0.82	0.016	80.0
	98	0.64–0.84	0.77	0.029	88.6
Leaf cholorophyll content (SPAD)	101	35.3–53.65	45.9	3.04	87.5
PHENO-score 1	87	37.00–51.50	43.06	3.99	66.2
PHENO-score 2	100	37.00–75.00	59.49	10.3	69.3
Leaf rolling (LR)	99	1.00–8.00	4.45	1.44	40.4
Dry biomass (ton/ha)	105	1.9–3.7	2.6	0.29	63.5

**Figure 4:**
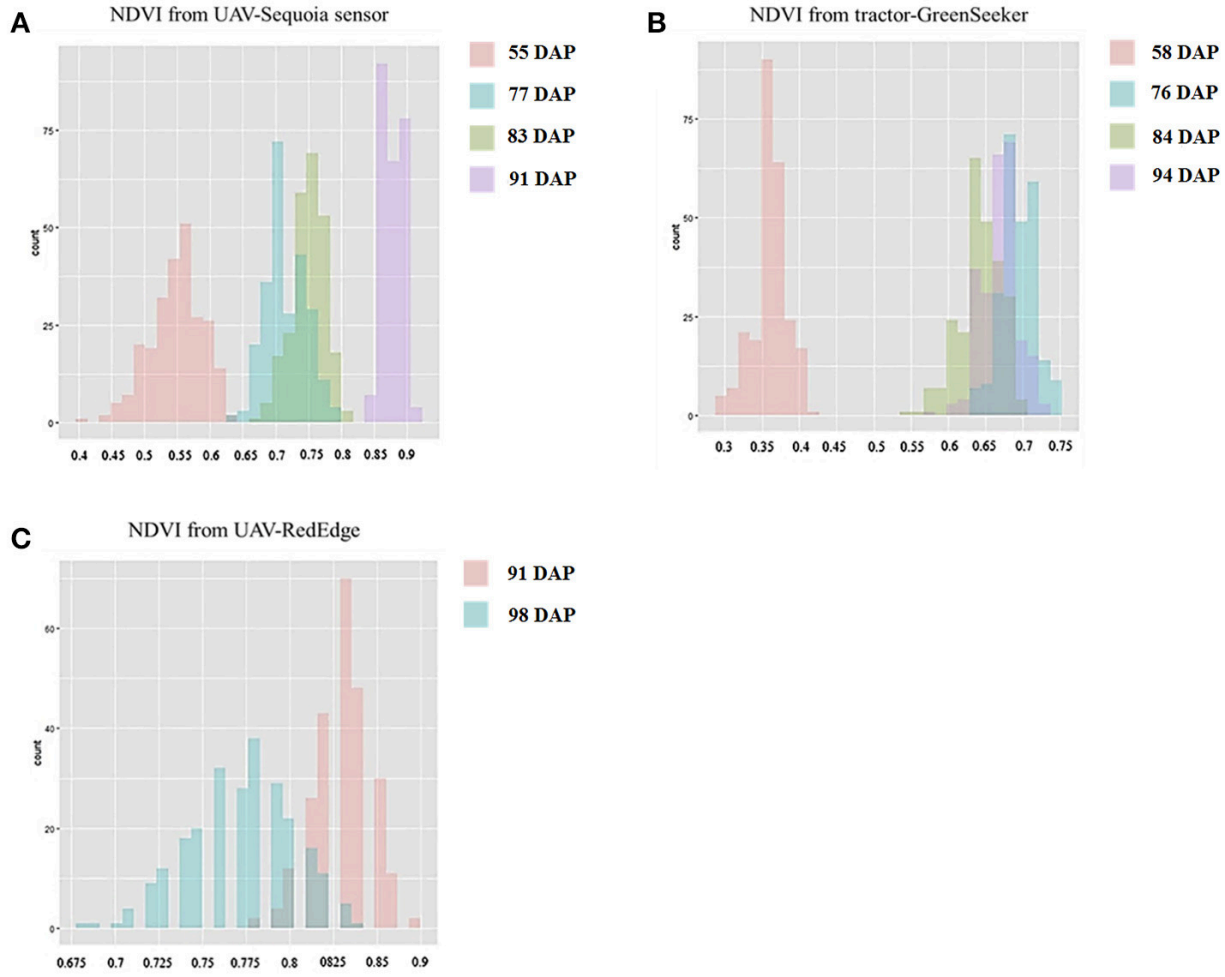
Histograms for NDVI-UAV-Sequoia **(A)**, NDVI-tractor-GreenSeeker **(B)**, and NDVI-UAV-RedEdge **(C)** at different days after planting (DAP).

The NDVI data collected with the GreenSeeker showed distributions with lower mean values compared to the UAV-derived data and, most importantly, reached the plateau already at 6 March (76 DAP) (Figure 3). Similarly to NDVI-UAV-Sequoia, the mean values progressively increased from 16 February to 24 March (55 DAP to 91 DAP). NDVI on 58 DAP averaged 0.36 and on 76 DAP reached 0.64, considered as the plateau for this platform (Figure 4). Table 3 reports Pearson's correlation coefficients among NDVI consecutive measurements, separately for UAV-Sequoia and tractor-GreenSeeker sensors.

**Table 3:** Broad-sense heritability and Pearson correlation coefficient for NDVI by UAV-Sequoia and tractor-GreenSeeker platforms on different days after planting (DAP).

**NDVI-UAV-Sequoia**	***h^*2*^*(%)**	**55**	**77**	**83**	**91**
55	77.2	1	–	–	–
77	83.2	0.747^***^	1	–	–
83	88.5	0.562^***^	0.859^***^	1	–
91	87.3	0.291^***^	0.555^***^	0.782^***^	1
**NDVI-Tractor-GreenSeeker**	***h***^***2***^**(%)**	**58**	**76**	**84**	**94**
58	61.1	1	–	–	–
76	66.3	0.661^***^	1	–	–
84	66.9	0.535^***^	0.869^***^	1	–
94	67.5	0.142^*^	0.415^***^	0.590^***^	1

*** *P < 0.001*,

* *0.01 < P < 0.05*.

Correlations reached medium to high values only for measurements taken at consecutive DAP, and were lower for non-consecutive DAP. Table 4 shows the correlations between UAV-Sequoia and tractor-GreenSeeker at comparable DAP. The correlations were all highly significant and ranged from 0.42 to 0.61 (*P* < 0.01), with the latter observed for the two measurements taken on 91 and 94 DAP.

**Table 4:** Pearson correlation coefficient for NDVI between UAV-Sequoia and tractor-GreenSeeker platforms on different days after planting (DAP).

**NDVI**	**Tractor-GreenSeeker**				
	**DAP**	**58**	**76**	**84**	**94**
UAV-Sequoia	55	0.506^***^	0.469^***^	0.384^***^	0.189^**^
	77	0.467^***^	0.507^***^	0.376^***^	0.243^***^
	83	0.357^***^	0.435^***^	0.423^***^	0.383^***^
	91	0.105^***^	0.219^**^	0.397^***^	0.614^***^

*** *P < 0.001*,

** *0.001 < P < 0.01*.

### Leaf chlorophyll content (SPAD), leaf rolling (LR), soil moisture, RWC, and dry biomass

Leaf chlorophyll content (SPAD) and leaf rolling (LR) as assessed under terminal drought stress conditions showed a normal distribution (Supplementary Figure 3). SPAD measurements ranged from 35.3 to 53.7 with an average of 46.0 while leaf rolling had an average of 4.45. Repeatability values were equal to 0.88 for SPAD, 0.40 for LR and 0.64 for dry biomass (Table 2). RWC results show that the cessation of irrigation on March 16 resulted in progressively lower leaf RWC for the four tested varieties (Supplementary Figure 4). In addition, soil moisture data on volumetric basis ranged from 7.1 to 13.8% indicating high levels of drought stress.

Dry biomass showed a normal distribution with an average of 2.61 ton ha^−1^. A positive correlation was observed between dry biomass and NDVI from aerial and tractor platforms with Pearson correlation coefficients ranging from 0.32 (91 and 94 DAP) to 0.53 (83 and 84 DAP) (Supplementary Figure 5).

### Effect of phenology-relevant loci on NDVI

Association testswere performed to investigate the effect of known phenology-relevant loci on the target traits (records of phenological stage and NDVI repeated measurements) (Table 5). *PPD-A1* had the strongest effect on phenology score, followed by *FT-7A* and *PPD-B1*. The photoperiod sensitive allele *PPD-A1-452* (Bentley et al., 2011), against all photoperiod-sensitive alleles had the strongest effects on phenology-score and NDVI measurements with –Log *P*-values equal to 9.69 and 12.16 for the two phenological scores and –Log *P*-values ranging from 2.46 to 7.52 for ground-based NDVI. The photoperiod-insensitive allele *PPD-A1-380* showed only a mildly significant effect compared to the insensitive allele *PPD-A1-452*. Also *FT-7A* showed significant effects on phenological scores and on both UAV- and ground-based NDVI on 91, 94, and 98 DAP. *PPD-B1* showed mild effects on phenology scores only, while *VRN-A1* had no effect on any of the drought-adaptive traits. In addition, *Rht-B1b* had a significant effect on dry biomass with –Log *P*-value equal to 3.14. The phenology-relevant loci did not affect the manually scored SPAD. Based on these results, the loci relevant for phenology/plant development were used as covariates in GWAS analysis for NDVI traits.

**Table 5:** Significance and associated effect for major loci known to affect phenology and plant height *PPD-A1*-452 (sensitivity vs. insensitivity), *PPD-A1*-380/290 (insensitivity alleles), *PPD-B1* (copy number variation polymorphism), *Rht-B1* (RhtB1b semi-dwarfism allele) and *FT-7A* (indel in promoter region) on phenology score (17.3.2017 and 30.3.2017), NDVI and dry biomass on different days after planting (DAP).

**Traits**	**DAP**	***PPD-A1-452***		***PPD-A1-380/290***		***PPD-B1***		***Rht-B1***		***FT-7A***	
		**–Log *P***	**Effect**	**–Log *P***	**Effect**	**–Log *P***	**Effect**	**–Log *P***	**Effect**	**–Log *P***	**Effect**
NDVI-UAV-Sequoia	91	**6.40^a^**	−9a^b^							**3.45**	−9a
NDVI-tractor-GreenSeeker	76							2.99	20a		
	84	2.46	−9a					2.79	10a		
	94	**7.52**	−9a							**4.11**	−1a
NDVI-UAV-RedEdge	91	**3.08**	−7a								
	98	**6.11**	−21a							**3.31**	−1a
PHENO-score1	87	**12.16**	8.65			2.76	2.05			**6.15**	2.71
PHENO-score2	100	**9.69**	3.79	2.12	3.57	2.56	8.65			**6.78**	7.46
Dry biomass	105							**3.14**	0.24		

a GWAS significance P < 0.0001 (corresponding to Bonferroni P 0.05 multiple test significance threshold) correspond to a bold underlined font, 0.0001 < P < 0.001 to a bold font and 0.001 < P < 0.01 to a regular font;

b *Effect: a = E−03*.

### GWAS for NDVI, dry biomass, leaf chlorophyll content (SPAD), leaf rolling, and phenology score

A total of 55 single NDVI QTLs were detected for the UAV-Sequoia platform on 55, 77, 83 and 91 DAP (detailed results reported in Supplementary Table 6), while for the similar DAP (58, 76, 84, and 94) the tractor-mounted platform identified 41 QTLs, about 25% fewer than with the UAV platform (Supplementary Tables 6, 7). In total, 28 QTLs were identified exclusively with the UAV-Sequoia platform while 15 QTLs were uniquely detected the tractor-mounted platform. When overlapping QTLs across platforms and GSs were considered as single identities, a total of 46 unique NDVI QTLs were identified (Supplementary Table 12). MLM-Q+K analysis detected 17 out of 46 unique NDVI QTLs on chromosomes 1A, 1B, 2B, 4A, 4B, 5A, 6A, 6B and 7A (Supplementary Table 13).

As to NDVI-UAV-Sequoia, the global *R*^2^ of multiple QTL models ranged from 24.2% on 77 DAP (6 QTLs) to 89.6% on 91 DAP (22 QTLs), as shown in Supplementary Table 6. For NDVI-tractor-GreenSeeker at the same growth stages, the global *R*^2^ of multiple QTL models ranged from 15.1% on 76 DAP (11 QTLs) to 64.7% on 94 DAP (16 QTLs). Notably, 19 of the 46 unique NDVI QTLs were consistently detected by both Sequoia-UAV and tractor-mounted platforms (41.30%, Supplementary Table 8). A common feature of both platforms was that the number of detectable NDVI QTLs and the global *R*^2^ of multiple QTL models sharply increased from 55–77 DAP (14 QTLs for UAV-Sequoia and 9 QTLs for tractor-GreenSeeker) to 76–94 DAP (41 QTLs for UAV-Sequoia and 32 QTLs for tractor-GreenSeeker), in coincidence with and/or after anthesis. Twelve QTLs (52% of all 23 QTLs) were detected by both platforms for 55–77 DAP and 41 of 73 NDVI QTLs (56%) were detected at 76–94 DAP.

As expected from the medium to low correlation value, NDVI QTLs were detected at each of the four DAP herein considered. Specific QTLs were found particularly on 76–77 DAP and 91–94 DAP. Table 6 reports the QTLs, commonly detected over at least two of the following inter-related traits: NDVI-UAV-Sequoia, NDVI-tractor-GreenSeeker, leaf chlorophyll content (SPAD), and dry biomass. A strong *per se* QTL influencing all eight NDVI measurements, SPAD and dry biomass was identified on chromosome 2B (*QNDVI.ubo-2B.1*), positioned at 5.9 cM on the tetraploid consensus map of Maccaferri et al. (2015a). *R*^2^-values for this QTL were 5.38% for NDVI-UAV-Sequoia (91 DAP), 6.29% for SPAD, and 5.67% for dry biomass. Importantly, the confidence interval of this QTL did not overlap with that of *PPD-B1* (mapped at 51.3 cM on chromosome 2B of the consensus map) and can thus be considered as a valuable constitutive *per se* QTL affecting NDVI from the vegetative stage under well-watered conditions up to late-milk grain filling under water-deficit conditions. Additional QTLs consistently detected for NDVI, SPAD and dry biomass mapped on chromosomes 4A and 4B (*QNDVI.ubo-4A.2* and *QNDVI.ubo-4B.1*), with the latter closely mapping to the well-known *RhtB1b* locus. At least nine additional QTLs on chromosomes 1A, 2B, 3A, 4B, 5B, 6B, 7A, and 7B (*QNDVI.ubo-1A.1, QNDVI.ubo-2B.1, QNDVI.ubo-2B.4, QNDVI.ubo-3A.1, QNDVI.ubo-4B.1, QNDVI.ubo-5B.4, QNDVI.ubo-6B.6, QNDVI.ubo-7A.4*, and *QNDVI.ubo-7B.1*) affected NDVI concomitantly with both SPAD and dry biomass (chr. 1A, 2B, 4B, 5B, 6B, and 7B) or dry biomass only (chr. 2B, 3A, and 7A), suggesting that these QTLs affected biomass accumulation during the fast-growing stage or during the remobilization/translocation phases. In all cases, eight out of nine QTLs had no effects on phenology, hence suggesting *per se* effects on NDVI unrelated to growth stage.

**Table 6:** NDVI GWAS-QTLs for UAV-Sequoia (DAP: 55, 77, 83, and 91) and Tractor-GreenSeeker platforms (DAP: 58, 76, 84, and 94), leaf chlorophyll content (SPAD) (101 DAP) and dry biomass (105 DAP), commonly detected for at least two traits. QTL significance, tagging-marker *R*^2^-value and co-localization with previously known NDVI QTLs are reported.

**QTL**	**Marker**	**Position**	**NDVI UAV-Sequoia**				**NDVI Tractor-GreenSeeker**				**SPAD**	**Dry biomass**	**NDVI QTL from literature**
		**cM^1^**	**55**	**77**	**83**	**91**	**58**	**76**	**84**	**94**	**101**	**105**	**QTL^2^**
*QNDVI.ubo-1A.1*	IWB72019	59.7			4.9^3^	4.6			4.74	3.92	5.03	5.13	e
*QNDVI.ubo-1B.2*	IWA8557	25.4							6.56				a,d
*QNDVI.ubo-1B.3*	IWA6917	67.6			5.99	**6.99**				6.99	**7.07**		
*QNDVI.ubo-2A.3*	IWB8175	107.0				**7.21**				3.90			
*QNDVI.ubo-2B.1*	IWB47560	5.9	3.41	4.53	4.82	5.38	5.35	**2.69**	**5.24**	5.37	**6.29**	5.67	
*QNDVI.ubo-2B.4*	wPt-2929	170.6			5.35			6.08				**6.3**	
*QNDVI.ubo-3A.1*	IWA5039	64.3			4.95				2.91			4.21	e,f
*QNDVI.ubo-3B.1*	IWB6062	2.4			4.84						**6.84**		e
*QNDVI.ubo-3B.3*	IWB8435	41.3				5.37				3.95	4.84		
*QNDVI.ubo-3B.4*	IWB24050	147.2							4.86		**8.45**		
*QNDVI.ubo-3B.5*	IWB22805	204.5	**4.2**				4.6				**6.79**		b
*QNDVI.ubo-4A.1*	IWB73476	22.2								7.83	4.63		b,f,g
*QNDVI.ubo-4A.2*	IWB60692	167.6	4.73	6.59	5.38	5.35			5.23	5.23	3.13		
*QNDVI.ubo-4B.1*	IWB70795	2.8	**8.01**	**5.02**	4.48	4.79		5.25			5.11	4.34	b
*QNDVI.ubo-4B.2*	IWB56078	32.9								3.00	**7.56**		b,d
*QNDVI.ubo-4B.3*	IWB72120	92.9				**5.98**				4.15	**6.63**		
*QNDVI.ubo-5A.3*	IWA3583	112.1				4.23						5.76	
*QNDVI.ubo-5B.1*	IWB73979	14.7				**5.89**				5.00	5.72		b,d,e
*QNDVI.ubo-5B.2*	IWB59038	48.9			4.75								c,d
*QNDVI.ubo-5B.3*	IWB54773	93.9									4.79		f
*QNDVI.ubo-5B.4*	wPt-0498	109				5.35					3.2	5.67	
*QNDVI.ubo-6B.6*	IWB45581	155.1			3.14	4.59			3.24	4.08	4.75	2.9	
*QNDVI.ubo-7A.2*	IWB44791	59.8				2.61				4.21	5.78		e
*QNDVI.ubo-7A.3*	IWB58341	131.3		4.90	**7.18**	**4.32**			4.63				
*QNDVI.ubo-7A.4*	IWB28063	181.8	3.30		4.37	**5.73**	**6.91**					4.61	
Global QTL model (*R*^2^, %)		-	45.0	24.2	59.5	89.6	15.4	15.1	42.1	64.7	97.2	64.0	

*The full list of GWAS-QTLs is reported in Supplementary Table 12*.

1 Chromosomes of QTL regions based on the tetraploid wheat consensus map (Maccaferri et al., 2015a);

2 a: (Shi et al., 2017); b: (Pinto et al., 2016); c: (Sukumaran et al., 2015); d: (Gao et al., 2015); e: (Li et al., 2014); f: (Bennett et al., 2012); g: (Pinto et al., 2010);

3 *Tagging-marker R^2^-values are reported. GWAS significance P < 0.0001 (corresponding to Bonferroni P 0.05 multiple test significance threshold) correspond to a bold underlined font, 0.0001 < P < 0.001 to a bold font and 0.001 < P < 0.01 to a regular font*.

Additionally, QTLs showed concurrent effects on NDVI (Table 7), and SPAD as well. However, for these QTLs no significant effects were detected on dry biomass, suggesting a prevalence of effects on chlorophyll content and/or senescence at the grain-filling stage without an appreciable impact on total biomass. Examples of these NDVI QTLs are *QNDVI.ubo-1B.3,-2A.1,-2A.2,-3A.2,-3B.1, -3B.3,-3B.4,-3B.5, -4A.1,-4A.2,-4B.2, -5B.1,-5B.3,-7A.2*, and *-7B.4*. Several QTLs affected only a single NDVI measurement and were therefore considered of marginal interest. UAV-RedEdge platform on 91 and 98 DAP identified 45 single QTLs for NDVI (Supplementary Table 9). A major *per se* NDVI locus (*QNDVI.ubo-6B.5*), not detectable by SPAD, was detected on 91 DAP (*R*^2^ = 8.43%) and on 98 DAP (*R*^2^ = 6.71%). However, this QTL was then ascertained to be coincident with a QTL for visual leaf rolling. The UAV-based platforms identified 13 common NDVI QTLs out of the 22 that were detected with at least one of the UAV-based platforms (Supplementary Table 9).

**Table 7:** Highly-significant GWAS-QTLs for NDVI (*P* < 0.0001) from UAV-RedEdge (DAP: 91 and 98), UAV-Sequoia (DAP: 55, 77, 83, and 91) and tractor-GreenSeeker (DAP: 58, 76, 84, and 94).

**Platform**	**DAP**	**QTL**	**Marker**	**Chr**.	**Position (cM)^1^**	**CI (cM)**	**Alleles**	**Effect**	**–Log *P***	***R^*2*^ (%)***
UAV-Sequoia	55	*QNDVI.ubo-4B.1*	IWB70795	4B	7.95	4.95-10.95	**A/**G^3^	9.9c^2^	4.89	8.01
	77	*QNDVI.ubo-4B.1*	IWB70795	4B	16	13-19	**A**/G	−6.2a	4.15	5.02
	83	*QNDVI.ubo-7A.3*	IWB58341	7A	124.1	121.1-127.1	**A**/G	−1.5b	4.57	7.18
	91	*QNDVI.ubo-1B.3*	IWA6917	1B	58.5	55.5-61.5	**A**/G	−5.9a	4.90	6.99
		*QNDVI.ubo-2A.1*	IWB34575	2A	46.6	43.6-49.6	**A**/G	0.09	4.43	7.21
UAV-RedEdge	91	*QNDVI.ubo-1B.3*	IWB31673	1B	59.1	56.1-62.1	**C**/T	−1.2b	4.07	5.89
		*QNDVI.ubo-6B.5*	IWB71546	6B	94.8	91.8-97.8	**A**/G	−1.5b	5.53	8.43
		*QNDVI.ubo-7A.4*	IWB28063	7A	181.8	178.8-184.8	**A**/G	0.01	4.62	6.83
	98	*QNDVI.ubo-1B.2*	IWB8612	1B	43.6	40.6–46.6	**G**/T	0.02	4.98	6.61
		*QNDVI.ubo-3B.3*	IWB1757	3B	32	29–35	**A**/C	−2.1b	4.89	6.47
		*QNDVI.ubo-6B.5*	IWB71546	6B	94.8	91.8–97.8	**A**/G	−2.3b	5.05	6.71
Tractor	58	*QNDVI.ubo-7A.4*	IWA8393	7A	183.2	180.2–186.2	**C**/T	0.03	4.11	6.91
	76	*QNDVI.ubo-2B.1*	IWB47560	2B	5.9	2.9–8.9	**C**/T	0.02	4.45	2.69
	84	*QNDVI.ubo-2B.1*	IWB47560	2B	5.9	2.9–8.9	**C**/T	0.01	4.29	5.24
	94	*QNDVI.ubo-4A.2*	wPt-3449	4A	161.5	158.5–164.5	**A**/T	0.01	5.35	4.25

*QTL regions were defined based on a confidence interval of ±3.0 cM from the map positions of the QTL tagging-SNPs*.

1 Chromosomes of QTL regions based on the tetraploid wheat consensus map (Maccaferri et al., 2015a);

2 Allele effect: a = E+01, b = E+02, and c = E−04;

3 *The estimate of the effect is referred to the allele highlighted in bold*.

Most of the NDVI-QTLs were detected from 76 to 94 DAP, with 12 out of 19 QTLs common to UAV- and tractor-mounted platforms, in contrast to only 3 QTLs detected on 55–58 DAP. As reported in Table 6, *R*^2^ of multiple QTL models for common NDVI QTLs showed a himasongher percentage of explained variance (PEV) for UAV-Sequoia than for NDVI-tractor-GreenSeeker. PEV was 45.0% on 55 DAP for UAV-Sequoia and considerably lower (15.4%) on 58 DAP for tractor-GreenSeeker. UAV-Sequoia and tractor-GreenSeeker showed a PEV of 59.5% (77 DAP) and 42.1% (76 DAP), respectively, while PEV was 89.6% (91 DAP) and 64.7% (94 DAP), respectively. In addition, PEV was equal to 73.9 and 91.8% for NDVI-UAV-RedEdge on 91 and 98 DAP, respectively.

A total of 39 significant QTLs were detected for SPAD, particularly on chromosomes 1A (*R*^2^ = 9.7%), 3B (*R*^2^ = 6.8%), 5A (*R*^2^ = 10.3%), 5B (*R*^2^ = 8.0%), and 7A (*R*^2^ = 9.3%). Out of the 39 SPAD-QTLs, a total of 22 loci (56%) overlapped between SPAD and NDVI. Among these 22 loci, 19 were not related to phenology. Selected SNPs associated to SPAD showed a very high global *R*^2^ of 97.2% (Table 6), most likely overestimated due to residual population structure effects not accounted for.

Leaf rolling (LR) was associated to nine significant QTLs with one with the largest effect on chromosome 3A (*R*^2^ = 6.34%), while selected SNPs associated to LR showed a global *R*^2^ of 36.0% (Supplementary Table 11). Co-localization was observed for LR and NDVI at *QNDVI.ubo-5B.2* and *QNDVI.ubo-6B.5*. In particular, the first locus was related to NDVI measured with both UAV- and ground-mounted platforms under drought stress. *QNDVI.ubo-6B.5* was even more interesting as it was strongly associated to NDVI signals from all three platforms and to LR but not SPAD nor biomass. In addition, LR co-mapped with leaf chlorophyll content (SPAD) measured on 101 DAP on chromosome 2B.

GWAS for dry biomass identified 19 significant QTLs (Supplementary Table 11) with the strongest effects shown by those on chromosomes 2B (*R*^2^ = 6.3%), 4B (*R*^2^ = 6.5%), 6A (*R*^2^ = 7.2%), and 7B (*R*^2^ = 6.4%). Nine of these QTLs were linked to NDVI (47% of UAV-detected QTLs) with *QNDVI.ubo-5A.3* (*R*^2^ = 5.8%) and *QNDVI.ubo-5B.4* (*R*^2^ = 5.7%) only detected with UAV-based platforms. Selected SNPs associated to dry biomass QTLs accounted for 64% of the phenotypic variance.

**Figure 5:**
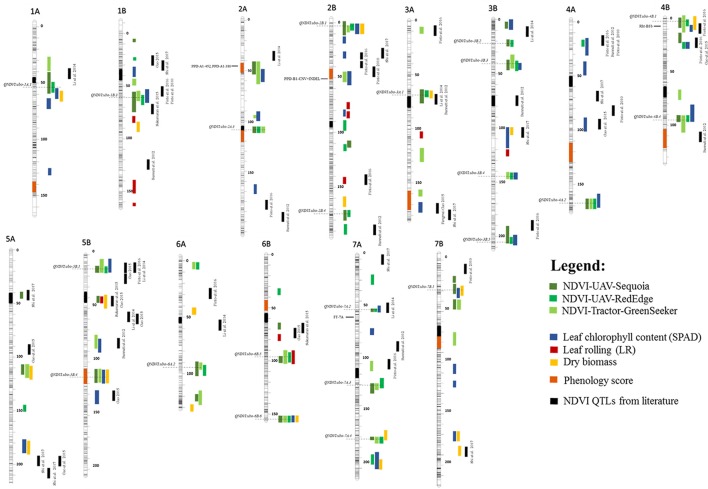
Chromosome position on the durum consensus map (Maccaferri et al., 2015a) of (i) QTLs identified in this study, (ii) previously mapped NDVI QTLs and (iii) main genes for phenology. NDVI QTLs significant for UAV-Sequoia are highlighted with a dark-green vertical bar, NDVI QTLs significant for UAV-RedEdge with a light-green bar, NDVI QTLs significant for tractor-GreenSeeker with a green-bar. QTLs highlighted with a yellow bar were significant for dry biomass, QTLs with a blue bar are significant for SPAD, QTLs with a red bar were significant for leaf rolling (LR) and QTLs indicated with an orange bar (shown directly on the chromosomes) are significant for phenology score. Black vertical bars indicate NDVI QTLs identified from the literature. Horizontal gray-dotted lines indicate the QTL peak positions.

The full QTL list for NDVI, dry biomass, leaf chlorophyll content (SPAD), LR and phenology scores is available in Supplementary Tables 6, 7, 11. For comparative analysis of our results with previously published work, all QTLs identified in this study were positioned on the tetraploid-consensus map assembled by Maccaferri et al. (2015a) and are reported in Figure 5, including also NDVI QTLs gathered from the literature, mainly identified in the hexaploid wheat germplasm with hand-held portable instruments such as the classic GreenSeeker. Notably, 23 of the 46 NDVI QTLs did not overlap with growth stage QTLs, hence suggesting a prevalence of effects on a *per se* basis.

Based on the results reported herein, eight QTL hotspots for NDVI and/or chlorophyll content (SPAD), leaf rolling (LR) and biomass unrelated to phenology were detected on chromosomes 1A (*QNDVI.ubo-1A.1*), 1B (*QNDVI.ubo-1B.3*), 2B (*QNDVI.ubo-2B.1*), 4B (*QNDVI.ubo-4B.1*), 5B (*QNDVI.ubo-5B*.1), 6B (*QNDVI.ubo-6B.5* and *QNDVI.ubo-6B.6*), and 7B (*QNDVI.ubo-7B.1*).

## Discussion

### NDVI measurements by UAV- and ground-based platforms

To our best knowledge, this study is the first to report on the use of UAV-based NDVI remote sensing for GWAS analysis in crops and to compare the results to those obtained using a ground-based platform. We compared two UAV- and one ground-based platforms to search for NDVI QTLs in a field trial first conducted under well-watered conditions until flowering, then followed by 2 weeks of progressively increasing water-deficit conditions that decreased leaf relative water content (RWC) to 53%. The rapid decrease in RWC after stopping irrigation was consequent to the high evaporative demand typical of the environment where the field trial was conducted. During the time interval from 16 to 31 March when irrigation was terminated and plants experienced an increasing water-deficit stress, the average mean daily and average maximum temperatures were 20.9 and 29.7°C, respectively while the average reference daily evapotranspiration using the standardized Penman-Monteith method was 5.41 mm.

It is well known that NDVI devices/platforms show different sensitivity features and, consequently, differ in their capacities to discriminate genotypes, specifically depending on the crop developmental stage and/or agronomic management (Marti et al., 2007; Cabrera-Bosquet et al., 2011; Christopher et al., 2016). Sensitivity of commonly used ground-based sensors such as GreenSeeker is maximum at early growth stages and then at the grain-filling/senescence stage while the sensitivity of UAV-based sensors, particularly for GWAS-QTL analysis, has not been assessed. Based on the known relationships of NDVI (as an integrative measure) with chlorophyll content and total plant/canopy biomass, a time-series of consecutive NDVI measurements were cross-referenced with flag leaf relative chlorophyll content (SPAD), leaf rolling and dry biomass data in order to identify the growth stage when NDVI and its relevant QTLs were most informative.

When compared to the two UAV-based platforms, NDVI-values collected with the ground-based platform plateaued earlier from 76 to 84 DAP, indicating its lower capacity to monitor plant biomass accumulation and leaf greenness during the reproductive stage of the wheat growth cycle. Additionally, UAV-mounted platforms allowed us to measure hundreds of plots in very short time, hence minimizing the confounding effects due to time-related environmental variation, which inevitably affect the results of studies conducted with ground-based platforms (Haghighattalab et al., 2016). Whether differences between the ground-based platform and UAV-based platforms are due to the means of locomotion or the nature of the sensors employed, they could not be assessed with these data.

NDVI has long been recognized for its ability to estimate crop biomass and grain yield (Lewis et al., 1998; Araus et al., 2001; Chuvieco Salinero, 2002) and this correlation becomes stronger when estimated with UAV platforms (Kyratzis et al., 2015). In our study, the two UAV-based platforms showed a markedly higher repeatability for NDVI measurements as compared to those collected with the ground-based platform. High repeatability, hence heritability, is critical to effectively identify and eventually clone QTLs (Tuberosa, 2012). Therefore, from a methodological perspective on the use of the aerial vs. ground-based HTPPs to detect QTL for NDVI, our results show the increased ability of the former, particularly under terminal drought stress, as shown by the considerably higher number of QTLs and overall *R*^2^-values detected with the UAV-based platforms. Accordingly, a recent study conducted in barley grown under 10 different nitrogen treatments has also shown an increased sensitivity of aerial vs. ground-based platforms to measure NDVI using RGB (conventional digital cameras), multispectral and thermal aerial imagery in combination with a matching suite of ground sensors (Kefauver et al., 2017). The relative benefits and comparison of UAV- and ground-based platforms remain to be empirically evaluated for other phenotypic variables beyond NDVI.

Notably, the NDVI measurements from UAV-RedEdge on 91 DAP showed a decrease in NDVI average values under water shortage, most likely consequent to the cumulative effects of senescence and drought stress severity. As reported by Peters et al. (2002), NDVI can indicate vegetation response to water stress and could be used as a proxy to evaluate drought effects (Kyratzis et al., 2015; Liu et al., 2016).

### GWAS analysis for NDVI and other drought-adaptive traits

It is known that spectrometers to measure NDVI and other vegetative indexes show different sensitivities. Consequently, sensors/platforms are also characterized by different capacity to discriminate among genotypes, depending on the developmental stage and/or agronomic management. In wheat, sensitivity of commonly used ground-based active sensors such as GreenSeeker is maximum at early growth stages while progressively decreasing approaching heading/anthesis (canopy closure) and then increasing again with the onset of the grain-filling/senescence phase (Marti et al., 2007; Christopher et al., 2016). While several GWAS studies have reported on the dissection of genetic inheritance of NDVI data collected with traditional ground-based sensors (as detailed below), no specific study has so far addressed the effectiveness of UAV-based sensors in providing NDVI scores suitable for QTL discovery. In our study, the UAV-based (Sequoia) NDVI data allowed for the identification of a considerably larger number (58%) of NDVI QTLs as compared to the ground-based platform (42%). Moreover, the use of the UAV-based platforms allowed us to increase the level of QTL significance and repeatability across growth stages. As expected, grain-filling stages appeared the most valuable for detecting NDVI-related genetic differences among genotypes (61.2% of which were identified at the grain-filling stage) in response to the progressive onset of senescence and drought stress related to the water-shortage treatment. Along this line, breeding strategies for enhancing drought tolerance are increasingly adopting remote-sensing of NDVI and other spectral technologies (Monneveux et al., 2012; Araus and Cairns, 2014; Ramya et al., 2016; Trapp et al., 2017).

Two main loci identified on chromosomes 2A (*R*^2^ from 6.40 to 7.21%) and 6B (*R*^2^ from 6.13 to 8.43%) were associated to NDVI QTLs as per UAV-based (Sequoia and RedEdge sensors) data during the water-stressed treatment (Supplementary Table 10). Based on the known relationships of NDVI (as integrative measure) with chlorophyll content and total plant biomass, NDVI measurements were cross-referenced to leaf chlorophyll content (SPAD) and dry biomass accumulation data. Among the 39 significant loci mapped for SPAD, 22 (56%) overlapped with NDVI QTLs from aerial and ground-based platforms.

### QTL hotspots for NDVI and other drought-related proxy traits

The major loci known to influence photoperiod, vernalization, flowering time, and plant height (Milner et al., 2016) significantly affected phenology score, NDVI, leaf rolling and dry biomass. In particular, *PPD-A1* and *FT-7A* influenced phenology score, UAV- and ground-based NDVI, especially under water-deficit stress. The strong effect on phenology score and adaptation of *PPD-A1* allelic variants is well documented (Snape et al., 2001) while the effects of variants at *PPD-B1* (copy number variation) and at *FT-7A* have been less explored. *PPD-A1* (452-bp allele) influenced UAV-based NDVI on 91 and 98 DAP as well as ground-based NDVI on 84 and 94 DAP, while *FT-7A* influenced UAV-based NDVI on 91 and 98 DAP as well as NDVI-tractor-GreenSeeker on 94 DAP (Table 5). Accounting for the effects (as covariates) of these major loci in the GWAS mixed model allowed us to markedly increase the power of QTL detection while providing more accurate estimates of their effects and identifying QTLs influencing drought-adaptive traits on a *per se* basis. After covariance analysis based on the molecular genotypes of accessions at the major *PPD, VRN*, and *FT* loci, six QTLs were still found to influence both NDVI and phenology score (Zadoks system). This notwithstanding, our study also highlighted the presence of eight hotspot QTLs affecting NDVI and/or chlorophyll content (SPAD), leaf rolling (LR), biomass and/or visual response to water shortage independently from phenology, further supported by co-location with NDVI QTLs reported in bread wheat.

The leaves of many important cereal crops (maize, rice, sorghum, and wheat) show a tendency to roll up into a cylinder in response to drought conditions and then unroll when leaf water balance improves (Sirault et al., 2015). Apart from mutant genetic stocks showing a constitutively high leaf rolling (LR), this trait in cultivated wheat germplasm is associated with leaf water loss and thus provides a proxy of drought stress over a certain degree of relative water loss. In this regard, the negative relationship observed between NDVI and LR, particularly evident with subgroup S1 (ancient Italian accessions) which showed the lowest NDVI (98 DAP) and the highest LR-values, suggests that modern durum wheat varieties for Mediterranean countries have been selected for both enhanced chlorophyll content and improved drought-response.

### Comparative analysis with other QTL studies in wheat

Although recent studies have identified significant NDVI QTLs in cereals (Pinto et al., 2010, 2016; Bennett et al., 2012; Li et al., 2014; Gao et al., 2015; Sukumaran et al., 2015; Shi et al., 2017; Figure 5), none of these studies deployed UAV-mounted cameras to collect multi-spectral images.

Among the five NDVI QTLs detected by Pinto et al. (2010) in elite Seri/Babax recombinant inbred lines (RILs) at vegetative and grain-filling stages, two overlapped with our QTLs on chromosomes 1B and 4A for ground-based NDVI. More recently, Pinto et al. (2016) detected the major QTL for NDVI at the vegetative stage in Seri/Babax wheat mapping population on chromosome 1B and other NDVI loci on chromosomes 1A, 2A, 4A, 5B, and 7A, all of which overlapped with each locus of this study, except for the QTL on chromosome 2A.

In particular, the NDVI QTL on chromosome 1B is of great interest, since it showed the highest LOD score and percentage explained variance (PEV) in Seri/Babax (Pinto et al., 2016) and it has been consistently detected across three NDVI phenotyping methods in our experiment, within a coincident confidence interval of <10 cM. Most notably, this QTL did not affect phenology. Therefore, this chromosome region represents an important hotspot for NDVI and leaf greenness and is a good candidate for marker-assisted selection as well as positional cloning (Salvi and Tuberosa, 2015). Studying the inheritance of this region in tetraploid wheat and eventually cloning the underlining functional polymorphism would also be a good complement toward the dissection of drought-adaptive traits in hexaploid wheat, in view of the simplified genetics of tetraploid wheats and, particularly, the recent assembly of a high-quality assembly in emmer wheat (*T. turgidum* ssp. *dicoccum* Schrank; Avni et al., 2017), the tetraploid progenitor of both durum and bread wheat.

Bennett et al. (2012) identified four significant loci for NDVI in RAC875/Kukri doubled-haploid population under heat and drought treatments and two of those overlapped with our QTLs for UAV-based NDVI on chromosomes 2B and 5B. Gao et al. (2015) reported NDVI QTLs in the Chinese Wheat Cross Zhou 8425b/Chinese Spring at anthesis and at 10 days post-anthesis, eight of which co-mapped with NDVI QTLs detected in this study and, in particular, a strong overlapping was identified on chromosomes 3B and 5B. Additionally, Li et al. (2014) detected NDVI QTLs in bread wheat (Jingdong 8/Aikang 5) overlapping with QTLs on chromosomes 1A, 3A, 3B, 5B, and 7A also identified in the present work. Additionally, according to the markers shared with the tetraploid consensus map (Maccaferri et al., 2015a), two significant NDVI QTLs were identified on chromosomes 1B and 5B at 13 and 7 cM, respectively, from the QTLs previously identified by Sukumaran et al. (2015) for NDVI at vegetative and grain-filling stages using GreenSeeker portable sensors on spring hexaploid wheat lines. According to Kyratzis et al. (2017), there is a close association between NDVI and leaf/canopy greenness in durum wheat. Moreover, SPAD provides an estimation of grain yield (Islam et al., 2014; Monostori et al., 2016) and grain protein concentration (Le Bail et al., 2005). As reported by Kyratzis et al. (2015), NDVI represents also a proxy for biomass and the efficient application of this technology in large breeding programs has become the next challenge. We identified 19 significant GWAS QTLs for dry biomass, nine of which (47.3%) co-mapped with NDVI from both aerial and ground-based platforms, hence confirming the usefulness of this vegetation index for predicting final wheat biomass (Marti et al., 2007; Pantazi et al., 2016). Notably, nine of these loci on chromosomes 1B, 2B, 3B, 4B, 6A, 6B, and 7B overlapped with QTLs for field thousand grain weight (TGW) and/or grain yield (GY) from data published in Maccaferri et al. (2011) and reanalyzed for GWAS based on the same SNP platform (Maccaferri et al., 2016) considered herein. In addition *QNDVI.ubo.5A.3* and *QNDVI.ubo.5B.4* were linked to both dry biomass and NDVI captured only from aerial platforms with a *R*^2^ of 5.76 and 5.67%, respectively (Table 6). Among the four main QTLs mapped for LR on chromosomes 1B, 3A, 3B, and 6B, the last one overlapped with the LR QTL reported by Peleg et al. (2009) in durum wheat × wild emmer RIL evaluated under drought stress.

In summary, eight QTL hotspots for NDVI and/or chlorophyll content (SPAD), leaf rolling (LR) and biomass unrelated to phenology were detected on chromosomes 1A (*QNDVI.ubo-1A.1*), 1B (Q*NDVI.ubo-1B.3*), 2B (*QNDVI.ubo-2B.1*), 4B (*QNDVI.ubo-4B.1*), 5B (*QNDVI.ubo-5B.1*), 6B (*QNDVI.ubo-6B.5* and *QNDVI.ubo-6B.6*) and 7B (*QNDVI.ubo-7B.1*). Notably, *QNDVI.ubo-2B.1, QNDVI.ubo-4B.1* and *QNDVI.ubo-6B.6* overlapped with QTLs for TGW and/or GY (Maccaferri et al., 2016).

## Conclusions

This study compared NDVI field phenotyping based on the emerging UAV-based platforms vs. the standard ground-based methods targeting an elite durum wheat collection suitable for GWAS analysis and representative of global durum breeding. The results reported herein demonstrated the great potential and effectiveness of both fixed-wing and multi-rotor UAV-based platforms to gather rapid, precise, and detailed NDVI measurements, which in turn considerably improved trait repeatability estimates, QTL identification and considerably increasing the portion of phenotypic variation accounted for by the multiple-QTL models. NDVI phenotypes and NDVI QTLs were cross-referenced by parallel leaf greenness (SPAD) and final biomass evaluation. The durum panel proved informative for the identification of QTLs for NDVI, SPAD, LR, and biomass. Strong effect NDVI QTLs were consistently detected across phenotyping platforms, with concomitant QTL effects on SPAD, LR and/or biomass. One major *per se* NDVI QTL detected on chromosome 1B (*QNDVI.ubo-1B.4*) across the three NDVI phenotyping platforms and for SPAD co-mapped in a 10-cM interval with a major NDVI QTL described in the CIMMYT spring hexaploid wheat germplasm. Therefore, this QTL is worth considering for further characterization as well as positional cloning. Moreover, three additional *per se* NDVI QTLs were detected across measurements, consistently expressed from the end of fast-growth stage on 91 DAP (*QNDVI.ubo-2B.1, QNDVI.ubo-4A.2* and *QNDVI.ubo-4B.1*) in addition to several specific NDVI QTLs were also detected, particularly for the grain-filling drought-stressed stages. Importantly, our results demonstrate that UAV-based platforms allow phenotypic data to be collected in high-throughput and with precision capable of discerning genetic differences to facilitate the detection of QTLs for drought-adaptive traits.

## Author contributions

MN and PA-S provided overall coordination of the field experiments including manual, ground, and aerial phenotyping. JW, MN, RW, MM, and RT designed the experiment. GC, JW, and MN conducted the field and laboratory measurements. RW and AF managed the UAV workflow including photogrammetry and zonal statistics. PA-S and JW managed ground-based data workflows. GC and MM conducted genotyping experiments. GC and MM analyzed the data, interpreted the results, and wrote the manuscript, under the supervision of RT and with contributions from all the other authors.

### Conflict of interest statement

The authors declare that the research was conducted in the absence of any commercial or financial relationships that could be construed as a potential conflict of interest.

## Footnotes

^1^Minitab Statistical Software release 9. Minitab Inc., 3081 Enterprise Drive, State College, PA 16801-3008.

## References

[B1] Andrade-Sanchez P. Gore M. A. Heun J. T. Thorp K. R. Carmo-Silva A. E. French A. N. (2013). Development and evaluation of a field-based high-throughput phenotyping platform. Funct. Plant Biol. 41, 68–79. 10.1071/FP13126 32480967

[B2] Araus J. L. Cairns J. (2014). Field high-throughput phenotyping: the new crop breeding frontier. Trends Plant Sci. 19, 52–61. 10.1016/j.tplants.2013.09.008 24139902

[B3] Araus J. L. Casadesus J. Bort J. (2001). Recent tools for the screening of physiological traits determining yield, in Application of Physiology in Wheat Breeding, eds Reynolds M. P. Ortiz-Monasterio J. I. McNab A. (Mexico: CIMMYT), 59–77.

[B4] Avni R. Nave M. Barad O. Baruch K. Twardziok S. O. Gundlach H.. (2017). Wild emmer genome architecture and diversity elucidate wheat evolution and domestication. Science 357, 93–97. 10.1126/science.aan0032 28684525

[B5] Barrett J. C. Fry B. Maller J. Daly M. J. (2005). Haploview: analysis and visualization of LD and haplotype maps. Bioinformatics 21, 263–265. 10.1093/bioinformatics/bth457 15297300

[B6] Barrs H. D. (1968). Determination of water deficits in plant tissues, in Water Deficits and Plant Growth, ed Kozolvski T. T. (New Delhi: Academic Press), 235–368.

[B7] Bennett D. Reynolds M. Mullan D. Izanloo A. Kuchel H. Langridge P.. (2012). Detection of two major grain yield QTL in bread wheat (*Triticum aestivum* L.) under heat, drought and high yield potential environments. Theor. Appl. Genet. 125, 1473–1485. 10.1007/s00122-012-1927-2 22772727

[B8] Bentley A. R. Turner A. S. Gosman N. Leigh F. J. Maccaferri M. Dreisigacker S. (2011). Frequency of photoperiod-insensitive *Ppd-A1a* alleles in tetraploid, hexaploid and synthetic hexaploid wheat germplasm. Plant Breed. 130, 10–15. 10.1111/j.1439-0523.2010.01802.x

[B9] Berger G. L. Liu S. Hall M. D. Brooks W. S. Chao S. Muehlbauer G. J.. (2013). Marker-trait associations in Virginia Tech winter barley identified using genome-wide mapping. Theor. Appl. Genet. 126, 693–710. 10.1007/s00122-012-2011-7 23139143

[B10] Bort J. Casadesus J. Nachit M. M. Araus J. L. (2005). Factors affecting the grain yield predicting attributes of spectral reflectance indices in durum wheat: growing conditions, genotype variability and date of measurement. Int. J. Remote Sens. 26, 2337–2358. 10.1080/01431160512331337808

[B11] Bowman B. C. Chen J. Zhang J. Wheeler J. Wang Y. Zhao W. (2015). Evaluating grain yield in spring wheat with canopy spectral reflectance. Crop Sci. 55, 1881–1890. 10.2135/cropsci2014.08.0533

[B12] Bradbury P. J. Zhang Z. Kroon D. E. Casstevens T. M. Ramdoss Y. Buckler E. S. (2007). TASSEL: software for association mapping of complex traits in diverse samples. Bioinformatics 23, 2633–2635. 10.1093/bioinformatics/btm308 17586829

[B13] Cabrera-Bosquet L. Sánchez C. Rosales A. Palacios-Rojas N. Araus J. L. (2011). Near-Infrared Reflectance Spectroscopy (NIRS) assessment of delta O18 and nitrogen and ash contents for improved yield potential and drought adaptation in maize. J. Agric. Food Chem. 59, 467–474. 10.1021/jf103395z 21175211

[B14] Christopher J. T. Christopher M. J. Borrell A. K. Fletcher S. Chenu K. (2016). Stay-green traits to improve wheat adaptation in well-watered and water-limited environments. J. Exp. Bot. 67, 5159–5172. 10.1093/jxb/erw276 27443279 PMC5014159

[B15] Chuvieco Salinero E. (2002). Analisis de Imagenes: Extraccion de Informacion Tematica. Teledeteccion Ambiental. Barcelona: Ariel, La observacion de la tierra desde el espacio.

[B16] Duan T. Chapman S. C. Guo Y. Zheng B. (2017). Dynamic monitoring of NDVI in wheat agronomy and breeding trials using an unmanned aerial vehicle. Field Crops Res. 210, 71–80. 10.1016/j.fcr.2017.05.025

[B17] Evanno G. Regnaut S. Goudet J. (2005). Detecting the number of clusters of individuals using the software STRUCTURE: a simulation study. Mol. Ecol. 14, 2611–2620. 10.1111/j.1365-294X.2005.02553.x 15969739

[B18] Gao F. Wen W. Liu J. Rasheed A. Yin G. Xia X.. (2015). Genome-wide linkage mapping of QTL for yield components, plant height and yield-related physiological traits in the Chinese Wheat Cross Zhou 8425B/Chinese Spring. Front. Plant. Sci. 6:1099. 10.3389/fpls.2015.01099 26734019 PMC4683206

[B19] Haghighattalab A. González Pérez L. Mondal S. Singh D. Schinstock D. Rutkoski J.. (2016). Application of unmanned aerial systems for high throughput phenotyping of large wheat breeding nurseries. Plant Methods. 12:35. 10.1186/s13007-016-0134-6 27347001 PMC4921008

[B20] Holman F. H. Riche A. B. Michalski A. Castle M. Wooster M. J. Hawkesford M. J. (2016). High throughput field phenotyping of wheat plant height and growth rate in field plot trials using UAV based remote sensing. Remote Sens. 8:1031 10.3390/rs8121031

[B21] Islam M. R. Sham sul Haque K. M. Akter N. Abdul Karim M. (2014). Leaf chlorophyll dynamics in wheat based on SPAD meter reading and its relationship with grain yield. Sci. Agric. 4, 13–18. 10.15192/PSCP.SA.2014.4.1.1318

[B22] Ji L. Peters A. J. (2003). Assessing vegetation response to drought in the northern Great Plains using vegetation and drought indices. Remote Sens. Environ. 87, 85–98. 10.1016/S0034-4257(03)00174-3

[B23] Kefauver S. C. Vicente R. Vergara-Díaz O. Fernandez-Gallego J. A. Kerfal S. Lopez A.. (2017). Comparative UAV and field phenotyping to assess yield and nitrogen use efficiency in hybrid and conventional barley. Front. Plant. Sci. 8:1733. 10.3389/fpls.2017.01733 29067032 PMC5641326

[B24] Kelley C. P. Mohtadi S. Cane M. A. Seager R. Kushnir Y. (2015). Climate change in the Fertile Crescent and implications of the recent Syrian drought. Proc. Natl. Acad. Sci. U.S.A. 112, 3241–3246. 10.1073/pnas.1421533112 25733898 PMC4371967

[B25] Kyratzis A. C. Skarlatos D. P. Fotopoulos V. Vamvakousis V. F. Katsiotis A. (2015). Investigating correlation among NDVI index derived by unmanned aerial vehicle photography and grain yield under late drought stress conditions. Proc. Env. Sci. 29, 225–226. 10.1016/j.proenv.2015.07.284

[B26] Kyratzis A. C. Skarlatos D. P. Menexes G. C. Vamvakousis V. F. Katsiotis A. (2017). Assessment of vegetation indices derived by UAV imagery for durum wheat phenotyping under a water limited and heat stressed mediterranean environment. Front. Plant. Sci. 8:1114. 10.3389/fpls.2017.01114 28694819 PMC5483459

[B27] Labus M. P. Nielsen G. A. Lawrence R. L. Engel R. Long D. S. (2010). Wheat yield estimates using multi-temporal NDVI satellite imagery. Int. J. Remote Sens. 23, 4169–4180. 10.1080/01431160110107653

[B28] Langridge P. Reynolds M. P. (2015). Genomic tools to assist breeding for drought tolerance. Curr. Opin. Biotechnol. 32, 130–135. 10.1016/j.copbio.2014.11.027 25531270

[B29] Le Bail M. Jeuffroy M. H. Bouchard C. Barbottin A. (2005). Is it possible to forecast grain protein content and yield of several varieties from chlorophyll meter measurements? Eur. J. Agron. 23, 379–391. 10.1016/j.eja.2005.02.003

[B30] Letta T. Maccaferri M. Badebo A. Ammar K. Ricci A. Crossa J.. (2013). Searching for novel sources of field resistance to Ug99 and Ethiopian stem rust races in durum wheat via association mapping. Theor. Appl. Genet. 126, 1237–1256. 10.1007/s00122-013-2050-8 23429902

[B31] Lewis J. E. Rowland J. Nadeau A. (1998). Estimating maize production in Kenya using NDVI: some statistical considerations. Int. J. Remote Sens. 19, 2609–2617. 10.1080/014311698214677

[B32] Li X. Chen X. Xiao Y. Xia X. Wang D. He Z. (2014). Identification of QTLs for seedling vigor in winter wheat. Euphytica 198, 199–209. 10.1007/s10681-014-1092-6

[B33] Liu W. Maccaferri M. Bulli P. Rynearson S. Tuberosa R. Chen X.. (2017). Genome-wide association mapping for seedling and field resistance to *Puccinia striiformis* f. *sp. tritici* in elite durum wheat. Theor. Appl. Genet. 130, 649–667. 10.1007/s00122-016-2841-9 28039515

[B34] Liu Z. Li C. Zhou P. Chen X. (2016). A probabilistic assessment of the likelihood of vegetation drought under varying climate conditions across China. Sci. Rep. 6:35105. 10.1038/srep35105 27713530 PMC5054395

[B35] Lobos G. A. Matus I. Rodriguez A. Romero-Bravo S. Araus J. L. Del Pozo A. (2014). Wheat genotypic variability in grain yield and carbon isotope discrimination under Mediterranean conditions assessed by spectral reflectance. J. Integr. Plant Biol. 56, 470–479. 10.1111/jipb.12114 24118723

[B36] Lukina E. V. Stone M. L. Rann W. R. (1999). Estimating vegetation coverage in wheat using digital images. J. Plant Nutr. 22, 341–350. 10.1080/01904169909365631

[B37] Maccaferri M. El-Feki W. Nazemi G. Salvi S. Canè M. A. Colalongo M. C.. (2016). Prioritizing quantitative trait loci for root system architecture in tetraploid wheat. J. Exp. Bot. 67, 1161–1178. 10.1093/jxb/erw039 26880749 PMC4753857

[B38] Maccaferri M. Ricci A. Salvi S. Milner S. G. Noli E. Martelli P. L.. (2015a). A high-density, SNP-based consensus map of tetraploid wheat as a bridge to integrate durum and bread wheat genomics and breeding. Plant Biotechnol. J. 13, 648–663. 10.1111/pbi.12288 25424506

[B39] Maccaferri M. Sanguineti M. C. Demontis A. El Ahmed A. Garcia del Moral L. Maalouf F.. (2011). Association mapping in durum wheat grown across a broad range of water regimes. J. Exp. Bot. 62, 409–438. 10.1093/jxb/erq287 21041372

[B40] Maccaferri M. Zhang J. Bulli P. Abate Z. Chao S. Cantu D.. (2015b). A genome-wide association study of resistance to stripe rust (*Puccinia striiformis* f. sp*. tritici*) in a worldwide collection of hexaploid spring wheat (*Triticum aestivum* L.). G3 5, 449–465. 10.1534/g3.114.014563 25609748 PMC4349098

[B41] Madec S. Baret F. de Solan B. Thomas S. Dutartre D. Jezequel S.. (2017). High-throughput phenotyping of plant height: comparing unmanned aerial vehicles and ground LiDAR estimates. Front. Plant Sci. 8:2002. 10.3389/fpls.2017.02002 29230229 PMC5711830

[B42] Marti J. Bort J. Slafer G. A. Araus J. L. (2007). Can wheat yield be assessed by early measurements of NDVI? Ann. Appl. Biol. 150, 253–257. 10.1111/j.1744-7348.2007.00126.x

[B43] Mason R. E. Addison C. K. Babar A. Acuna A. Lozada D. Subramanuan N. (2018). Diagnostic markers for vernalization and photoperiod loci improve genomic selection for grain yield and spectral reflectance in wheat. Crop Sci. 58, 242–252. 10.2135/cropsci2017.06.0348

[B44] Milner S. G. Maccaferri M. Huang B. E. Mantovani P. Massi A. Frascaroli E.. (2016). A multiparental cross population for mapping QTL for agronomic traits in durum wheat (*Triticum turgidum* ssp. *durum*). Plant Biotechnol. J. 14, 735–748. 10.1111/pbi.12424 26132599 PMC11388855

[B45] Money D. Gardner K. Migicovsky Z. Schwaninger H. Zhong G. Y. Myles S. (2015). LinkImpute: fast and accurate genotype imputation for nonmodel organisms. G3 5, 2383–2390. 10.1534/g3.115.021667 26377960 PMC4632058

[B46] Monneveux P. Jing R. Misra S. C. (2012). Phenotyping for drought adaptation in wheat using physiological traits. Front. Physiol. 3:429. 10.3389/fphys.2012.00429 23181021 PMC3499878

[B47] Monostori L. Kadar B. Bauernhansl T. Kondoh S. Kumara S. Reinhart G. (2016). Cyber-physical systems in manufacturing. CIRP Ann. Manuf. Technol. 65, 621–641. 10.1016/j.cirp.2016.06.005

[B48] Ortiz R. Sayre K. D. Govaerts B. Gupta R. Subbarao G. V. Ban (2008). Climate change: can wheat beat the heat? Agric. Ecosyst. Environ. 126, 46–58. 10.1016/j.agee.2008.01.019

[B49] Pantazi X. E. Moshou D. Alexandridis T. Whetton R. L. Mouazen A. M. (2016). Wheat yield prediction using machine learning and advanced sensing techniques. Comput. Electron. Agric. 121, 57–65. 10.1016/j.compag.2015.11.018

[B50] Pauli D. Andrade-Sanchez P. Carmo-Silva A. E. Gazave E. French A. N. Heun J.. (2016). Field-based high-throughput plant phenotyping reveals the temporal patterns of quantitative trait loci associated with stress-responsive traits in cotton. G3 6, 865–879. 10.1534/g3.115.023515 26818078 PMC4825657

[B51] Peleg Z. Fahima T. Krugman T. Abbo S. Yakir D. Korol A. B. (2009). Genomic dissection of drought resistance in durum wheat × wild emmer wheat recombinant inbred line population. Plant Cell Environ. 32, 758–779. 10.1111/j.1365-3040.2009.01956.x 19220786

[B52] Peters J. A. Elizabeth A. Shea W. Lel J. I. Vliia A. Hayes M. (2002). Drought monitoring with NDVI-based standardized vegetation index. Photogramm. Eng. Remote Sens. 68, 71–75.

[B53] Pinto R. S. Lopes M. S. Collins N. C. Reynolds M. P. (2016). Modelling and genetic dissection of staygreen under heat stress. Theor. Appl. Genet. 129:2055. 10.1007/s00122-016-2757-4 27545985 PMC5069319

[B54] Pinto R. S. Reynolds M. P. Mathews K. L. McIntyre C. L. Olivares-Villegas J. J. Chapman S. C. (2010). Heat and drought adaptive QTL in a wheat population designed to minimize confounding agronomic effects. Theor. Appl. Genet. 121, 1001–1021. 10.1007/s00122-010-1351-4 20523964 PMC2938441

[B55] Price A. L. Zaitlen N. A. Reich D. Patterson N. (2010). New approaches to population stratification in genome-wide association studies. Nat. Rev. Genet. 11, 459–463. 10.1038/nrg2813 20548291 PMC2975875

[B56] Pritchard J. K. Stephens M. Donnelly P. (2000). Inference of population structure using multilocus genotype data. Genetics 155, 945–959. 10835412 10.1093/genetics/155.2.945 PMC1461096

[B57] Ramya P. Singh G. P. Jain N. Singh P. K. Pandey M. K. Sharma K. (2016). Effect of recurrent selection on drought tolerance and related morpho-physiological traits in bread wheat. PLoS ONE 11:e0156869 10.1371/journal.pone.0156869 27300000 PMC4907515

[B58] Rexroad C. E. Vallejo R. L. (2009). Estimates of linkage disequilibrium and effective population size in rainbow trout. BMC Genet. 10:83. 10.1186/1471-2156-10-83 20003428 PMC2800115

[B59] Reynolds M. Tuberosa R. (2008). Translational research impacting on crop productivity in drought-prone environments. Curr. Opin. Plant. Biol. 11, 171–179. 10.1016/j.pbi.2008.02.005 18329330

[B60] Reynolds M. Dreccer F. Trethowan R. (2007). Drought-adaptive traits derived from wheat wild relatives and landraces. J. Exp. Bot. 58, 177–186. 10.1093/jxb/erl250 17185737

[B61] Saitou N. Nei M. (1987). The neighbour-joining method: a new method for reconstructing phylogenetic trees. Mol. Biol. Evol. 4, 406–426. 3447015 10.1093/oxfordjournals.molbev.a040454

[B62] Salvi S. Tuberosa R. (2015). The crop QTLome comes of age. Curr. Opin. Biotechnol. 32, 179–185. 10.1016/j.copbio.2015.01.001 25614069

[B63] Shakoor N. Lee S. Mockler T. C. (2017). High throughput phenotyping to accelerate crop breeding and monitoring of diseases in the field. Curr. Opin. Plant Biol. 38, 184–192. 10.1016/j.pbi.2017.05.006 28738313

[B64] Shi S. Azam F. I. Li H. Chang X. Li B. Jing R. (2017). Mapping QTL for stay-green and agronomic traits in wheat under diverse water regimes. Euphytica 213, 1–19. 10.1007/s10681-017-2002-5

[B65] Sirault X. R. Condon A. G. Wood J. T. Farquhar G. D. Rebetzke G. J. (2015). “Rolled-upness”: phenotyping leaf rolling in cereals using computer vision and functional data analysis approaches. Plant Methods. 11:52. 10.1186/s13007-015-0095-1 26583042 PMC4650205

[B66] Snape J. Butterworth K. Whitechurch E. Worland A. J. (2001). Waiting for fine times: genetics of flowering time in wheat. Euphytica 119, 185–190. 10.1023/A:1017594422176

[B67] Sukumaran S. Dreisigacker S. Lopes M. Chavez P. Reynolds M. P. (2015). Genome-wide association study for grain yield and related traits in an elite spring wheat population grown in temperate irrigated environments. Theor. Appl. Genet. 128, 353–363. 10.1007/s00122-014-2435-3 25490985

[B68] Tattaris M. Reynolds M. P. Chapman S. C. (2016). A direct comparison of remote sensing approaches for high-throughput phenotyping in plant breeding. Front. Plant Sci. 7:1131. 10.3389/fpls.2016.01131 27536304 PMC4971441

[B69] Trapp J. J. Urrea C. A. Zhou J. Khot L. R. Sankaran S. Miklas P. N. (2017). Selective phenotyping traits related to multiple stress and drought response in dry bean. Crop Sci. 56, 1460–1472. 10.2135/cropsci2015.05.0281

[B70] Tuberosa R. (2012). Phenotyping for drought tolerance of crops in the genomics era. Front. Physiol. 3:347. 10.3389/fphys.2012.00347 23049510 PMC3446691

[B71] Tucker C. J. (1979). Red and photographic infrared linear combinations for monitoring vegetation. Remote Sens. Environ. 8, 127–150. 10.1016/0034-4257(79)90013-0

[B72] Wang S. Wong D. Forrest K. Allen A. Chao S. Huang B.. (2014). Characterization of polyploid wheat genomic diversity using a high-density 90,000 single nucleotide polymorphism array. Plant Biotechnol. J. 12, 787–796. 10.1111/pbi.12183 24646323 PMC4265271

[B73] Yang D. Liu Y. Cheng H. Chang L. Chen J. Chai S.. (2016). Genetic dissection of flag leaf morphology in wheat (*Triticum aestivum* L.) under diverse water regimes. BMC Genetics 17:94. 10.1186/s12863-016-0399-9 27352616 PMC4924301

[B74] Yousfi S. Márquez A. J. Betti M. Araus J. L. Serret M. D. (2016). Gene expression and physiological responses to salinity and water stress of contrasting durum wheat genotypes. J. Integr. Plant Biol. 58, 48–66. 10.1111/jipb.12359 25869057

[B75] Yu J. Pressoir G. Briggs W. H. Vroh Bi I. Yamasaki M. Doebley J. F.. (2006). A unified mixed-model method for association mapping that accounts for multiple levels of relatedness. Nat. Genet. 38, 203–208. 10.1038/ng1702 16380716

[B76] Zadoks J. C. Chang T. T. Konzak C. F. (1974). A decimal code for the growth stages of cereals. Weed Res. 14, 415–421. 10.1111/j.1365-3180.1974.tb01084.x

[B77] Zaman-Allah M. Vergara O. Araus J. L. Tarekegne A. Magorokosho C. Zarco-Tejada P. J. (2015). Unmanned aerial platform-based multispectral imaging for field phenotyping of maize. Plant Methods 11:35 10.1186/s13007-015-0078-2 26106438 PMC4477614

[B78] Zhang Z. Ersoz E. Lai C. Q. Todhunter R. J. Tiwari H. K. Gore M. A.. (2010). Mixed linear model approach adapted for genome-wide association studies. Nat. Genet. 42, 355–360. 10.1038/ng.546 20208535 PMC2931336

