# Corrigendum: Comparative Aerial and Ground Based High Throughput Phenotyping for the Genetic Dissection of NDVI as a Proxy for Drought Adaptive Traits in Durum Wheat

**DOI:** 10.3389/fpls.2018.01885

**Published:** 2018-12-24

**Authors:** Giuseppe E. Condorelli, Marco Maccaferri, Maria Newcomb, Pedro Andrade-Sanchez, Jeffrey W. White, Andrew N. French, Giuseppe Sciara, Rick Ward, Roberto Tuberosa

**Affiliations:** ^1^Department of Agricultural Sciences, University of Bologna, Bologna, Italy; ^2^Maricopa Agricultural Center, University of Arizona, Tucson, AZ, United States; ^3^US Arid Land Agricultural Research Center, USDA-ARS, Maricopa, AZ, United States

**Keywords:** *Triticum turgidum* L. subsp. *durum*, durum wheat, drought, high-throughput phenotyping, UAV, NDVI, GWAS, QTL

In the original article, we neglected to include the acknowledgment of the TERRA REF project, funded by the Advanced Research Projects Agency-Energy (ARPA-E), U.S. Department of Energy, under award number DE-AR0000594.

The authors apologize for this error and state that this does not change the scientific conclusions of the article in any way. The original article has been updated.

