# Supplementary material for: Comparative Aerial and Ground Based High Throughput Phenotyping for the Genetic Dissection of NDVI as a Proxy for Drought Adaptive Traits in Durum Wheat
Source: Front Plant Sci. 2018 Jun 26;9:893. doi: 10.3389/fpls.2018.00893 (PMC6028805; doi:10.3389/fpls.2018.00893)
Supplement: Supplementary file 8 [file Table_8.DOCX]

**Comparative Aerial and Ground Based High Throughput Phenotyping for the Genetic Dissection of NDVI as a Proxy for Drought Adaptive Traits in Durum Wheat**

*Giuseppe Emanuele Condorelli^1^, Marco Maccaferri^1*^, Maria Newcomb^2^, Pedro Andrade-Sanchez^2^, Jeffrey W. White^3^, Andrew N. French^3^, Giuseppe Sciara^1^, Rick Ward^2^ and Roberto Tuberosa^1^*

*^1^Department of Agricultural Sciences, University of Bologna, Italy, ^2^Maricopa Agricultural Center (MAC), University of Arizona, USA. ^3^US Arid-Land Agricultural Research Center, USDA-ARS, Maricopa, AZ, USA*

**Supplementary Table 8 |** List of GWAS-QTLs significantly associated with NDVI (p-value <0.0001) in common between UAV-Sequoia and tractor-GreenSeeker (A: 55-58 DAP, B: 76-77 DAP, C: 83-84 DAP and D: 91-94 DAP). The side significance and effect on phenology scores at two dates (PHENO1 and PHENO2), leaf angle (LA) and leaf rolling (LR) are also reported. The best P-value related to UAV-Sequoia are indicated as underlined. ***P-value <0.0001, **0.0001<P-value <0.001, *0.001<P-value <0.01.

| QTL | CI (cM) | NDVI  A | NDVI  B | NDVI  C | NDVI  D | SPAD | PHENO1 | PHENO2 | LR |
| --- | --- | --- | --- | --- | --- | --- | --- | --- | --- |
| *QNDVI.ubo-1A.1* | **56.7-62.7** |  |  | 3.31** | 3.44** | 3.42** |  |  |  |
| *QNDVI.ubo-1B.3* | **55.5.-61.5** |  |  |  | 4.9*** | 4.56*** |  |  |  |
| *QNDVI.ubo-2A.3* | **104-110** |  |  |  | 4.23*** |  | 5.09*** | 3.78** |  |
| *QNDVI.ubo-2B.1* | **2.9-8.9** | 3.69** | 4.45*** | 4.29*** | 3.93** | 4.13*** |  |  |  |
| *QNDVI.ubo-2B.4* | **167.6-173.6** |  |  | 3.57** |  |  |  |  |  |
| *QNDVI.ubo-3A.1* | **61.3-67.3** |  |  | 3.34** |  |  |  |  |  |
| *QNDVI.ubo-3B.2* | **19.3-25.3** |  |  | 3.28** |  |  |  |  |  |
| *QNDVI.ubo-3B.3* | **41.6-47.6** |  |  |  | 3.92** | 3.31** |  |  |  |
| *QNDVI.ubo-3B.5* | **191-197** | 4.83** |  |  |  |  |  |  |  |
| *QNDVI.ubo-4A.2* | **158.5-164.5** |  | 3.03** | 3.59** | 3.91** | 2.31* |  |  |  |
| *QNDVI.ubo-4B.1* | **0-5.8** |  | 4.15*** |  |  |  |  |  |  |
| *QNDVI.ubo-4B.3* | **89.9-95.9** |  |  |  | 4.29*** | 3.47** |  |  |  |
| *QNDVI.ubo-5B.1* | **11.7-17.7** |  |  |  | 4.24*** | 3.81** |  |  |  |
| *QNDVI.ubo-6A.2* | **90.4-96.4** |  |  |  | 3.71** |  |  |  |  |
| *QNDVI.ubo-6B.5* | **93.4-102.7** |  |  |  | 4.39*** |  |  |  | 3.71** |
| *QNDVI.ubo-6B.6* | **152.1-158.1** |  |  | 3.54** | 3.44** | 3.26** |  |  |  |
| *QNDVI.ubo-7A.2* | **56.8-62.8** |  |  |  | 3.09** | 3.84** |  |  |  |
| *QNDVI.ubo-7A.3* | **128.6-134.6** |  |  | 4.57*** |  |  |  |  |  |
| *QNDVI.ubo-7A.4* | **178.8-184.8** | 4.11*** |  |  |  | 5.01*** |  |  |  |
