# Supplementary material for: Comparative Aerial and Ground Based High Throughput Phenotyping for the Genetic Dissection of NDVI as a Proxy for Drought Adaptive Traits in Durum Wheat
Source: Front Plant Sci. 2018 Jun 26;9:893. doi: 10.3389/fpls.2018.00893 (PMC6028805; doi:10.3389/fpls.2018.00893)
Supplement: Supplementary file 12 [file Table_12.DOCX]

**Comparative Aerial and Ground Based High Throughput Phenotyping for the Genetic Dissection of NDVI as a Proxy for Drought Adaptive Traits in Durum Wheat**

*Giuseppe Emanuele Condorelli^1^, Marco Maccaferri^1*^, Maria Newcomb^2^, Pedro Andrade-Sanchez^2^, Jeffrey W. White^3^, Andrew N. French ^3^, Giuseppe Sciara^1^, Rick Ward^2^ and Roberto Tuberosa^1^*

*^1^Department of Agricultural Sciences, University of Bologna, Italy, ^2^Maricopa Agricultural Center (MAC), University of Arizona, USA. ^3^US Arid-Land Agricultural Research Center, USDA-ARS, Maricopa, AZ, USA*

**Supplementary Table 12 |** List of GWAS-QTLs (MLM + K + phenology-relevant loci covariates) significantly associated with NDVI for UAV-Sequoia (DAP: 55, 77, 83, 91) and Tractor-GreenSeeker (DAP: 58, 76, 84, 94) leaf chlorophyll content (DAP): 101 and dry biomass (DAP: 105) and commonly detected for at least two traits. QTL significance, tagging-marker *R^2^* values and co-localization with previously known NDVI QTLs are reported.

(1) High-density, SNP-based consensus map of tetraploid wheat (Maccaferri et al. 2015). (2) Days after planting (DAP) (3) a: Shi et al. 2017; b: Pinto et al. 2016; c: Sukumaran et al. 2016; d: Gao et al. 2015; e: Lin et al. 2014; f: Bennett et al. 2012; g: Pinto et al. 2010. (4) Tagging-marker R2 values are reported. P-value < 0.0001 correspond to a bold underlined font, 0.0001 < P-value <0.001 to a bold font and 0.001 < P-value <0.01 to a regular font.

| QTL | Marker | NDVI  UAV-Sequoia | | | | NDVI  Tractor-GreenSeeker | | | | SPAD | Dry biomass | NDVI QTL from literature |
| --- | --- | --- | --- | --- | --- | --- | --- | --- | --- | --- | --- | --- |
|  |  | **55 (2)** | **77** | **83** | **91** | **58** | **76** | **84** | **94** | **101** | **105** | **Co-mapping**  **QTLs (3)** |
| *QNDVI.ubo.1A.1* | **IWB72019(1)** |  |  | **4.9(4)** | **4.6** |  |  | 4.74 | 3.92 | **5.03** | **5.13** | e |
| *QNDVI.ubo.1B.1* | **IWB12157** |  |  | **4.67** |  |  |  |  |  |  |  |  |
| *QNDVI.ubo.1B.2* | **IWA8557** |  |  |  |  |  |  | **6.56** |  |  |  | a,d |
| *QNDVI.ubo.1B.3* | **IWA6917** |  |  | **5.99** | **6.99** |  |  |  | **6.99** | **7.07** |  |  |
| *QNDVI.ubo.2A.1* | **IWA2526** |  |  |  | **5.53** |  |  |  |  | **7.40** |  |  |
| *QNDVI.ubo.2A.2* | **IWB71685** |  |  |  |  |  |  |  |  | **7.45** |  |  |
| *QNDVI.ubo.2A.3* | **IWB8175** |  |  |  | **7.21** |  |  |  | **3.90** |  |  |  |
| *QNDVI.ubo.2B.1* | **IWB47560** | 3.41 | 4.53 | **4.82** | **5.38** | **5.35** | **2.69** | **5.24** | **5.37** | **6.29** | **5.67** |  |
| *QNDVI.ubo.2B.2* | **IWB5274** |  |  |  |  |  |  | **4.77** |  |  |  |  |
| *QNDVI.ubo.2B.3* | **IWB70242** |  |  |  |  |  |  | **4.66** |  |  |  |  |
| *QNDVI.ubo.2B.4* | **wPt-2929** |  |  | **5.35** |  |  | 6.08 |  |  |  | **6.30** |  |
| *QNDVI.ubo.2B.5* | **WPt-3378** |  |  |  |  | **5.01** |  |  |  |  |  | f |
| *QNDVI.ubo.3A.1* | **IWA5039** |  |  | **4.95** |  |  |  | 2.91 |  |  | **4.21** | e,f |
| *QNDVI.ubo.3A.2* | **IWB69546** |  |  |  |  |  |  |  |  | **4.44** |  |  |
| *QNDVI.ubo.3B.1* | **IWB6062** |  |  | **4.84** |  |  |  |  |  | **6.84** |  | e |
| *QNDVI.ubo.3B.2* | **IWB64404** |  |  | **5.29** |  |  |  | 4.72 |  |  |  |  |
| *QNDVI.ubo.3B.3* | **IWB8435** |  |  |  | **5.37** |  |  |  | **3.95** | **4.84** |  |  |
| *QNDVI.ubo.3B.4* | **IWB24050** |  |  |  |  |  |  | **4.86** |  | **8.45** |  |  |
| *QNDVI.ubo.3B.5* | **IWB22805** | **4.2** |  |  |  | **4.6** |  |  |  | **6.79** |  | b; |
| *QNDVI.ubo.4A.1* | **IWB73476** |  |  |  |  |  |  |  | **7.83** | **4.63** |  | b,f,g; |
| *QNDVI.ubo.4A.2* | **IWB60692** | **4.73** | **6.59** | **5.38** | **5.35** |  |  | **5.23** | **5.23** | 3.13 |  |  |
| *QNDVI.ubo.4B.1* | **IWB70795** | **8.01** | **5.02** | **4.48** | **4.79** |  | **5.25** |  |  | **5.11** | **4.34** | b |
| *QNDVI.ubo.4B.2* | **IWB56078** |  |  |  |  |  |  |  | **3.00** | **7.56** |  | d,b |
| *QNDVI.ubo.4B.3* | **IWB72120** |  |  |  | **5.98** |  |  |  | **4.15** | **6.63** |  |  |
| *QNDVI.ubo.5A.1* | **IWB70406** | **5.11** |  |  |  |  |  |  |  |  |  | a |
| *QNDVI.ubo.5A.2* | **IWB14582** |  |  |  |  | **6.72** |  |  |  |  |  |  |
| *QNDVI.ubo.5A.3* | **IWA3583** |  |  |  | **4.23** |  |  |  |  |  | **5.76** |  |
| *QNDVI.ubo.5A.4* | **IWB26265** |  |  |  |  |  |  |  | **4.19** |  |  |  |
| *QNDVI.ubo.5B.1* | **IWB73979** |  |  |  | **5.89** |  |  |  | **5.00** | **5.72** |  | b,d,e |
| *QNDVI.ubo.5B.2* | **IWB59038** |  |  | **4.75** |  |  |  |  |  |  |  | c,d |
| *QNDVI.ubo.5B.3* | **IWB54773** |  |  |  |  |  |  |  |  | **4.79** |  | f |
| *QNDVI.ubo.5B.4* | **WPt-0498** |  |  |  | **5.35** |  |  |  |  | 3.2 | **5.67** |  |
| *QNDVI.ubo.6A.1* | **IWA7288** |  |  |  |  |  | **3.33** |  |  |  |  |  |
| *QNDVI.ubo.6A.2* | **IWB42262** |  |  |  | 2.45 |  |  |  | **5.00** |  |  |  |
| *QNDVI.ubo.6A.3* | **IWB54807** |  |  |  | **3.94** |  |  |  |  |  |  |  |
| *QNDVI.ubo.6B.4* | **IWB35384** |  | **4.47** |  |  |  |  |  |  |  |  | c,d |
| *QNDVI.ubo.6B.5* | **IWB46951** |  |  |  | **6.13** |  |  |  | 3.12 |  |  |  |
| *QNDVI.ubo.6B.6* | **IWB45581** |  |  | **3.14** | **4.59** |  |  | **3.24** | 4.08 | **4.75** | 2.9 |  |
| *QNDVI.ubo.7A.1* | **IWB60043** |  |  |  |  |  |  |  | **4.04** |  |  |  |
| *QNDVI.ubo.7A.2* | **IWB44791** |  |  |  | 2.61 |  |  |  | **4.21** | **5.78** |  | e |
| *QNDVI.ubo.7A.3* | **IWB58341** |  | **4.90** | **7.18** | **4.32** |  |  | 4.63 |  |  |  |  |
| *QNDVI.ubo.7A.4* | **IWB28063** | 3.30 |  | **4.37** | **5.73** | **6.91** |  |  |  |  | **4.61** |  |
| *QNDVI.ubo.7B.1* | **IWB27459** |  |  |  | **5.2** |  |  |  |  | **5.70** | **6.41** |  |
| *QNDVI.ubo.7B.2* | **IWB73291** |  |  |  | **4.49** |  |  |  |  |  |  |  |
| *QNDVI.ubo.7B.3* | **IWA7589** |  |  |  | **4.16** |  |  |  |  |  |  |  |
| *QNDVI.ubo.7B.4* | **IWB9899** | **4.56** | **5.29** |  |  |  |  |  |  | **4.74** |  |  |
