# Supplementary material for: Comparative Aerial and Ground Based High Throughput Phenotyping for the Genetic Dissection of NDVI as a Proxy for Drought Adaptive Traits in Durum Wheat
Source: Front Plant Sci. 2018 Jun 26;9:893. doi: 10.3389/fpls.2018.00893 (PMC6028805; doi:10.3389/fpls.2018.00893)
Supplement: Supplementary file 13 [file Table_13.DOCX]

**Supplementary Table 13 |** List of GWAS-QTLs (MLM + Q + K + phenology-relevant loci covariates) significantly associated with NDVI for UAV-Sequoia (DAP: 55, 77, 83, 91) and Tractor-GreenSeeker (DAP: 58, 76, 84, 94). QTL significance, tagging-marker *R^2^* values and co-localization with previously known NDVI QTLs are reported.

(1) High-density, SNP-based consensus map of tetraploid wheat (Maccaferri et al. 2015). (2) Days after planting (DAP) (3) b: Pinto et al. 2016; c: Sukumaran et al. 2016; d: Gao et al. 2015; e: Lin et al. 2014; f: Bennett et al. 2012; g: Pinto et al. 2010. (4) Tagging-marker R2 values are reported. P-value < 0.0001 correspond to a bold underlined font, 0.0001 < P-value <0.001 to a bold font and 0.001 < P-value <0.01 to a regular font.

| QTL | Marker | NDVI  UAV-Sequoia | | | | NDVI  Tractor-GreenSeeker | | | | NDVI QTL from literature |
| --- | --- | --- | --- | --- | --- | --- | --- | --- | --- | --- |
|  |  | **55 (2)** | **77** | **83** | **91** | **58** | **76** | **84** | **94** | **Co-mapping**  **QTLs (3)** |
| *QNDVI.ubo.1A.1* | **IWB72053(1)** |  |  | **5.04(4)** | **4.31** |  |  |  |  | e |
| *QNDVI.ubo.1B.3* | **IWA7982** |  |  |  | **4.21** |  |  |  |  |  |
| *QNDVI.ubo.2B.1* | **IWB57438** |  |  |  |  |  | **5.64** | **4.79** |  |  |
| *QNDVI.ubo.2B.2* | **IWB9834** |  |  |  |  |  |  | **4.31** |  |  |
| *QNDVI.ubo.4A.1* | **IWB74418** |  |  |  |  |  | **4.44** |  | **4.94** | b,f,g; |
| *QNDVI.ubo.4A.2* | **wPt-7354** |  |  |  |  |  |  |  | **6.28** |  |
| *QNDVI.ubo.4B.1* | **IWB55598** | **6.34** | **6.53** |  |  |  | **5.63** |  |  | b |
| *QNDVI.ubo.4B.3* | **IWB12276** |  |  |  |  |  |  | **4.32** | **5.47** |  |
| *QNDVI.ubo.5A.2* | **IWB14582** |  |  |  |  | **5.25** |  |  |  |  |
| *QNDVI.ubo.5A.4* | **IWB1737** |  |  |  |  |  |  |  | **5.01** |  |
| *QNDVI.ubo.5B.4* | **IWB35312** | **5.05** |  |  |  |  |  |  |  |  |
| *QNDVI.ubo.6A.1* | **IWA7288** |  | **4.53** | **4.94** |  |  | **5.4** | **4.45** |  |  |
| *QNDVI.ubo.6A.2* | **IWB72393** |  |  |  |  |  |  |  | **5** |  |
| *QNDVI.ubo.6A.3* | **IWB24237** |  |  |  | **3.85** |  |  |  |  |  |
| *QNDVI.ubo.6B.4* | **IWB42809** |  | **5** |  |  |  |  |  |  | c,d |
| *QNDVI.ubo.6B.5* | **IWB46951** |  |  |  | **4.64** |  |  |  |  |  |
| *QNDVI.ubo.7A.3* | **IWB58341** |  |  | **6.36** | **5.3** |  |  |  |  |  |
