# Supplementary material for: Comparative Aerial and Ground Based High Throughput Phenotyping for the Genetic Dissection of NDVI as a Proxy for Drought Adaptive Traits in Durum Wheat
Source: Front Plant Sci. 2018 Jun 26;9:893. doi: 10.3389/fpls.2018.00893 (PMC6028805; doi:10.3389/fpls.2018.00893)
Supplement: Supplementary file 14 [file Presentation_1.PPTX]

## Slide 1
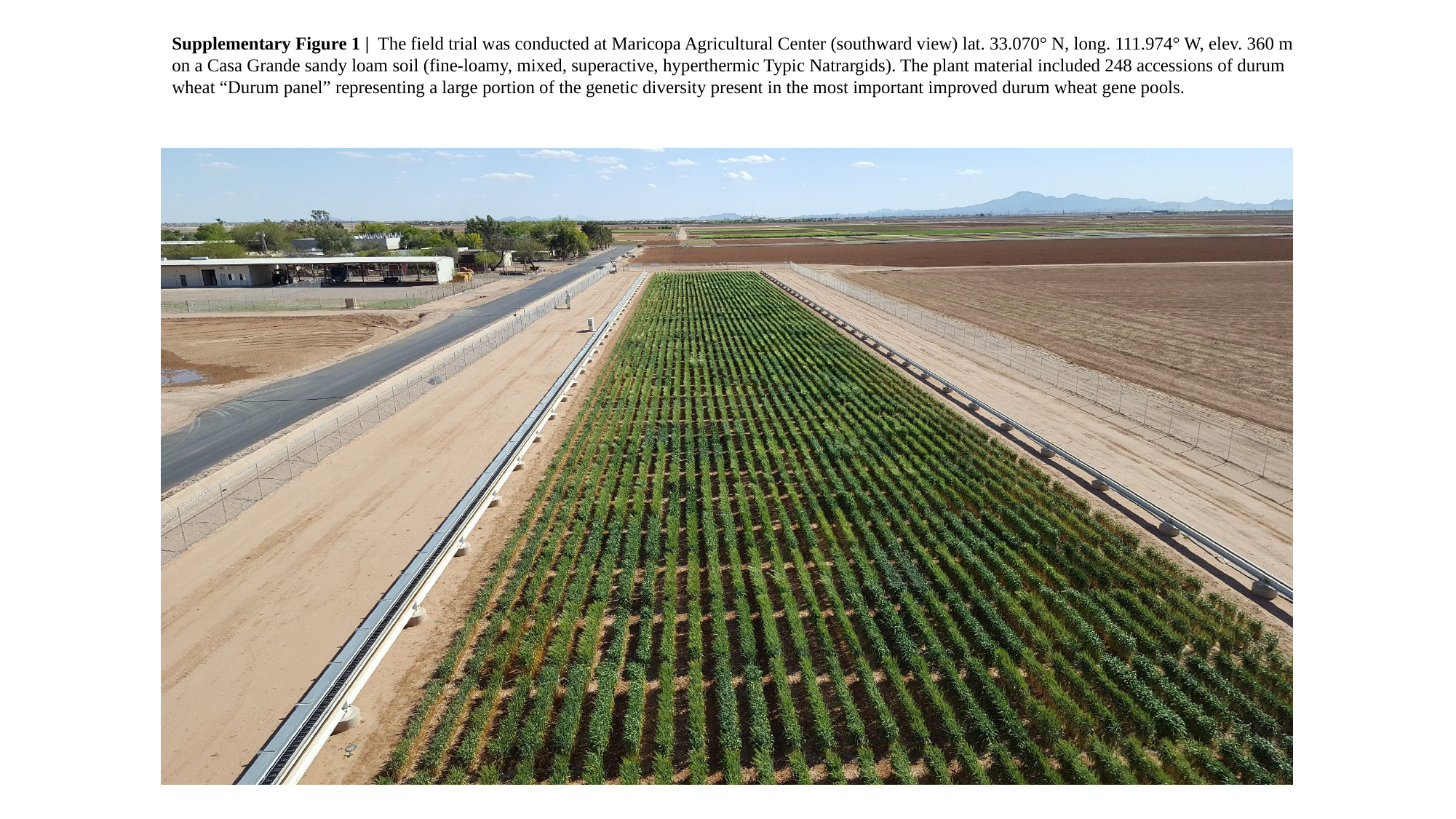

Supplementary Figure 1 |  The field trial was conducted at Maricopa Agricultural Center (southward view) lat. 33.070° N, long. 111.974° W, elev. 360 m on a Casa Grande sandy loam soil (fine-loamy, mixed, superactive, hyperthermic Typic Natrargids). The plant material included 248 accessions of durum wheat “Durum panel” representing a large portion of the genetic diversity present in the most important improved durum wheat gene pools.
