# Supplementary material for: Comparative Aerial and Ground Based High Throughput Phenotyping for the Genetic Dissection of NDVI as a Proxy for Drought Adaptive Traits in Durum Wheat
Source: Front Plant Sci. 2018 Jun 26;9:893. doi: 10.3389/fpls.2018.00893 (PMC6028805; doi:10.3389/fpls.2018.00893)
Supplement: Supplementary file 15 [file Presentation_2.PPTX]

## Slide 1
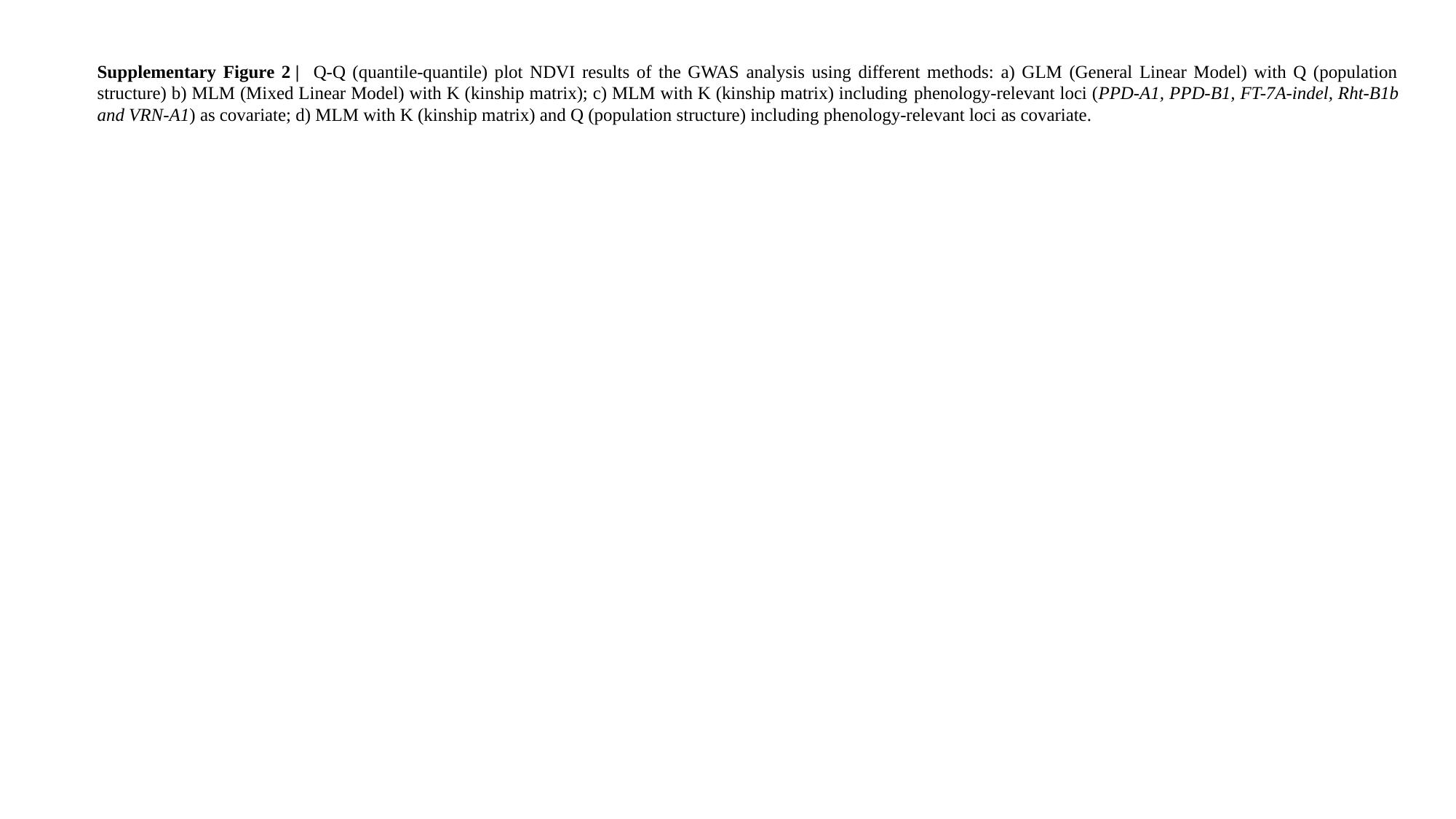

Supplementary Figure 2 | Q-Q (quantile-quantile) plot NDVI results of the GWAS analysis using different methods: a) GLM (General Linear Model) with Q (population structure) b) MLM (Mixed Linear Model) with K (kinship matrix); c) MLM with K (kinship matrix) including phenology-relevant loci (PPD-A1, PPD-B1, FT-7A-indel, Rht-B1b and VRN-A1) as covariate; d) MLM with K (kinship matrix) and Q (population structure) including phenology-relevant loci as covariate.

## Slide 2
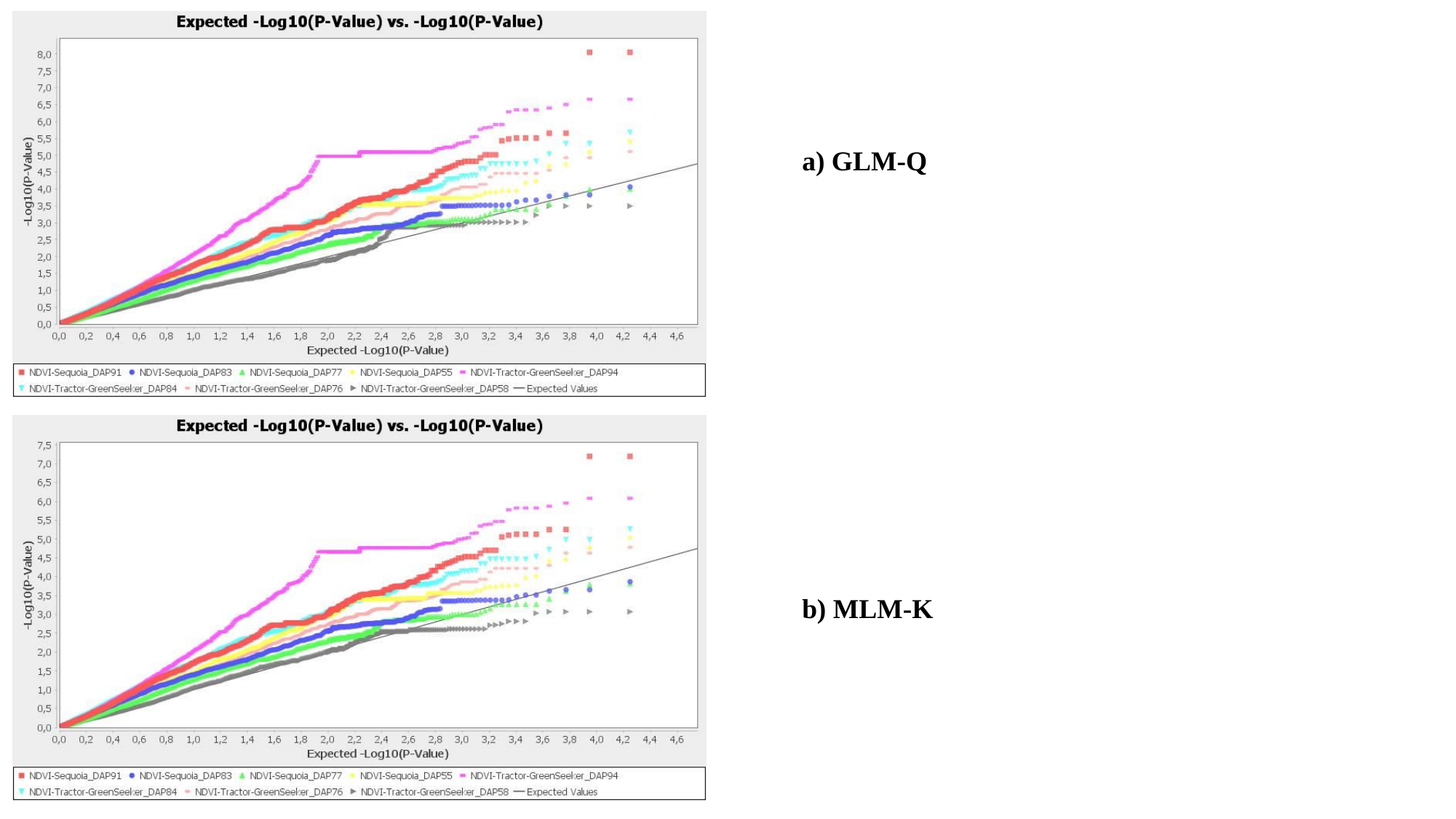

a) GLM-Q
b) MLM-K

## Slide 3
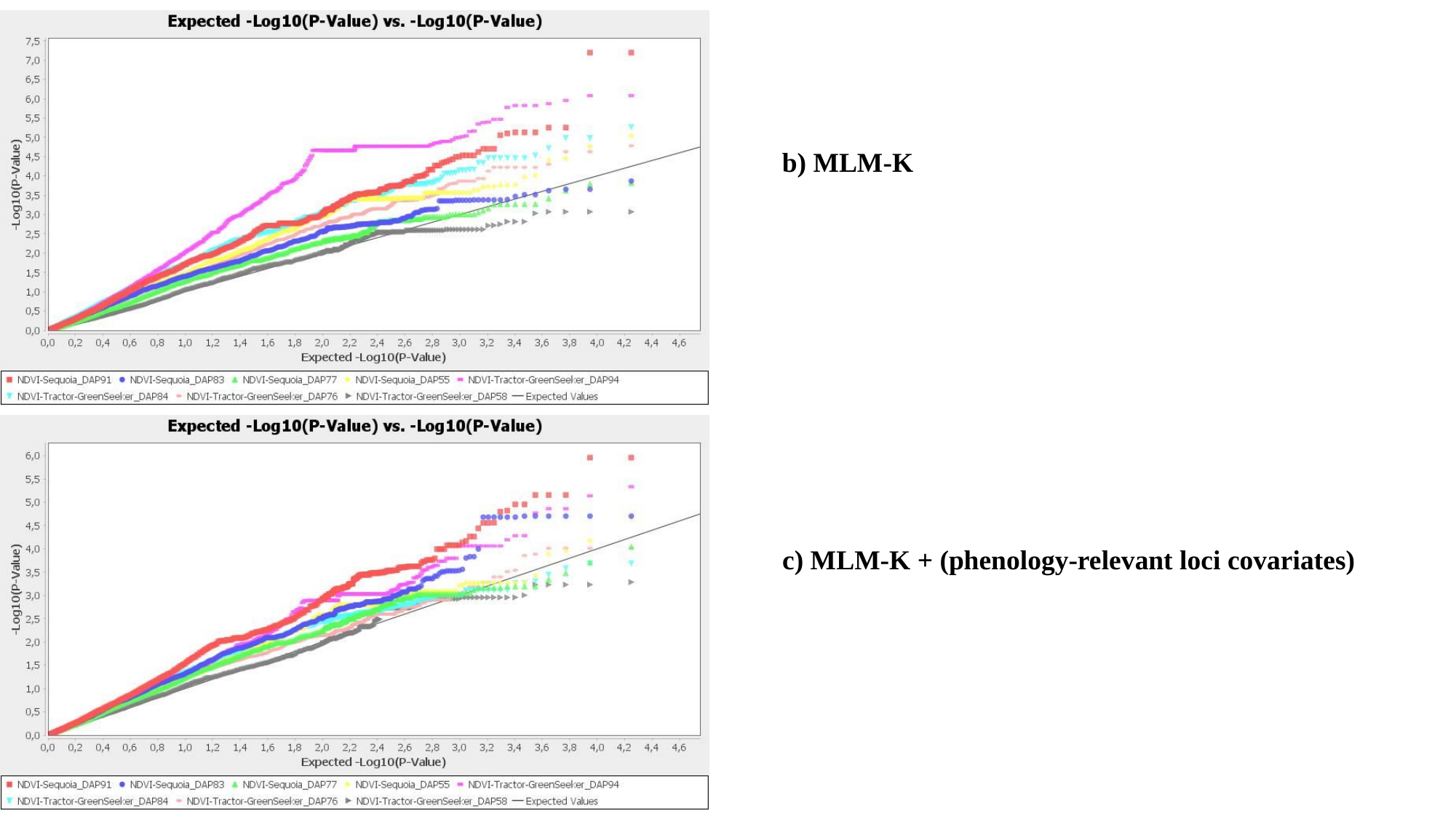

b) MLM-K
c) MLM-K + (phenology-relevant loci covariates)

## Slide 4
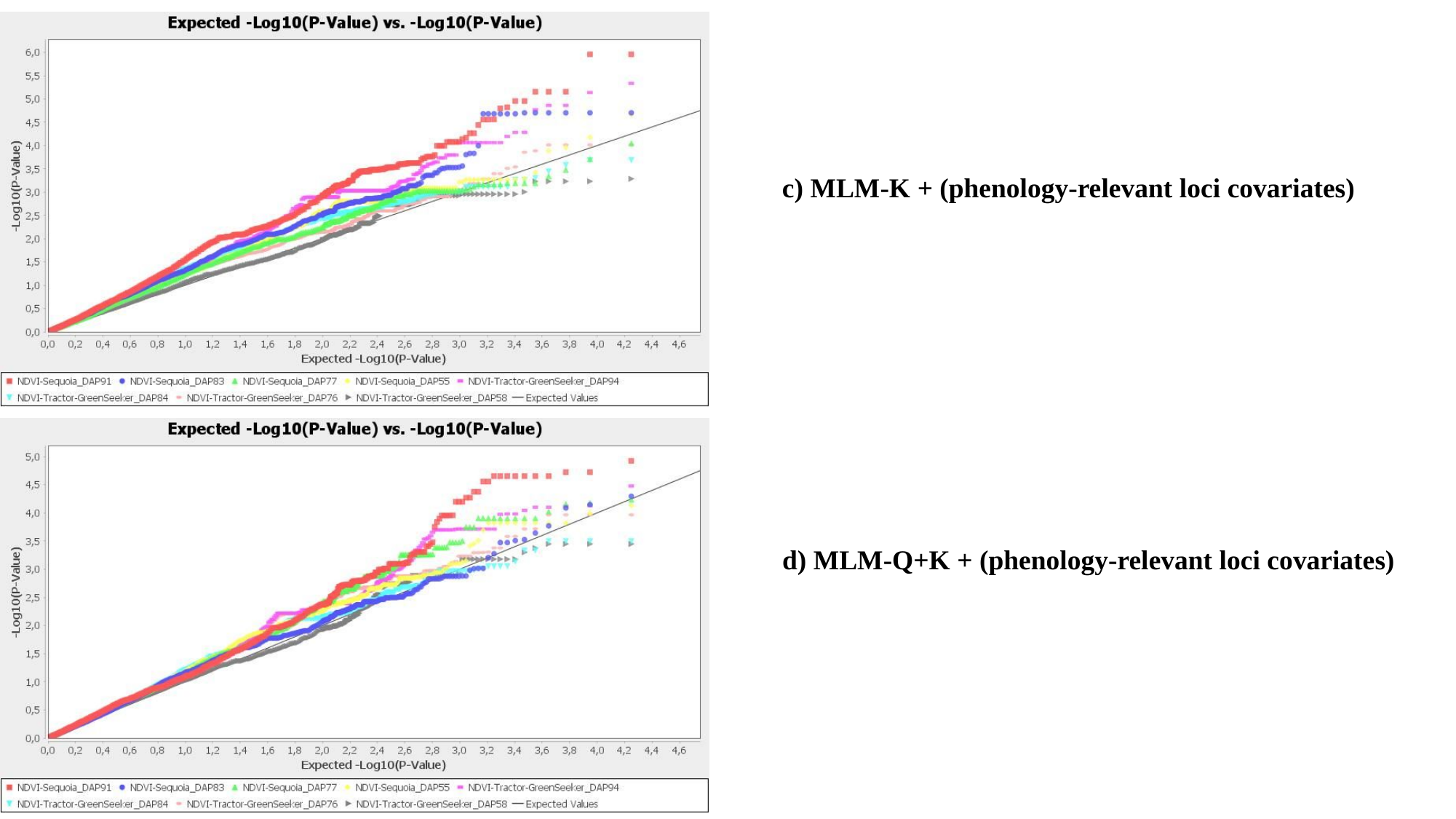

c) MLM-K + (phenology-relevant loci covariates)
d) MLM-Q+K + (phenology-relevant loci covariates)
