# Supplementary material for: Comparative Aerial and Ground Based High Throughput Phenotyping for the Genetic Dissection of NDVI as a Proxy for Drought Adaptive Traits in Durum Wheat
Source: Front Plant Sci. 2018 Jun 26;9:893. doi: 10.3389/fpls.2018.00893 (PMC6028805; doi:10.3389/fpls.2018.00893)
Supplement: Supplementary file 16 [file Presentation_3.PPTX]

## Slide 1
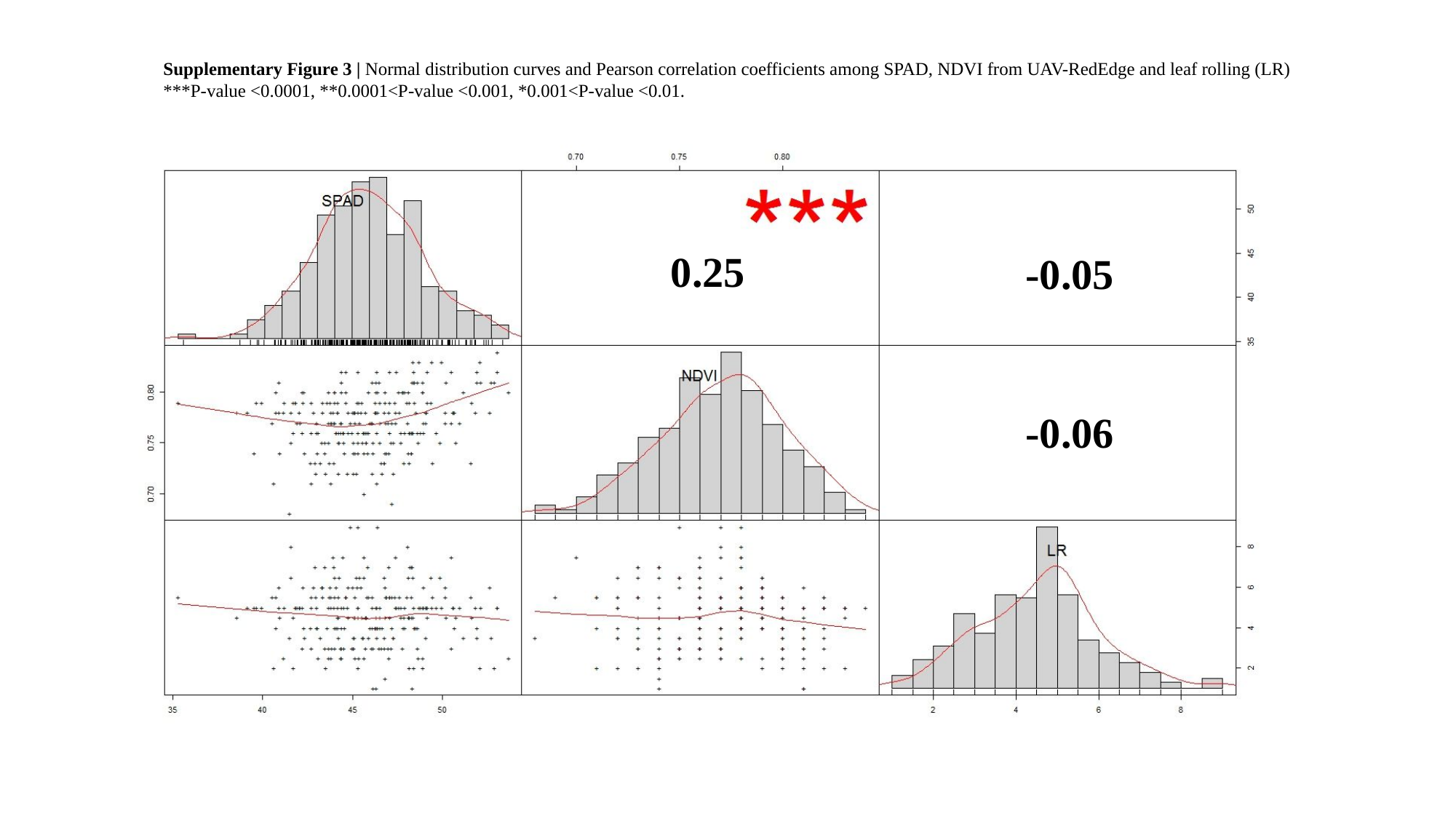

Supplementary Figure 3 | Normal distribution curves and Pearson correlation coefficients among SPAD, NDVI from UAV-RedEdge and leaf rolling (LR)
***P-value <0.0001, **0.0001<P-value <0.001, *0.001<P-value <0.01.
0.25
-0.05
-0.06
