# Supplementary material for: Comparative Aerial and Ground Based High Throughput Phenotyping for the Genetic Dissection of NDVI as a Proxy for Drought Adaptive Traits in Durum Wheat
Source: Front Plant Sci. 2018 Jun 26;9:893. doi: 10.3389/fpls.2018.00893 (PMC6028805; doi:10.3389/fpls.2018.00893)
Supplement: Supplementary file 17 [file Presentation_4.PPTX]

## Slide 1
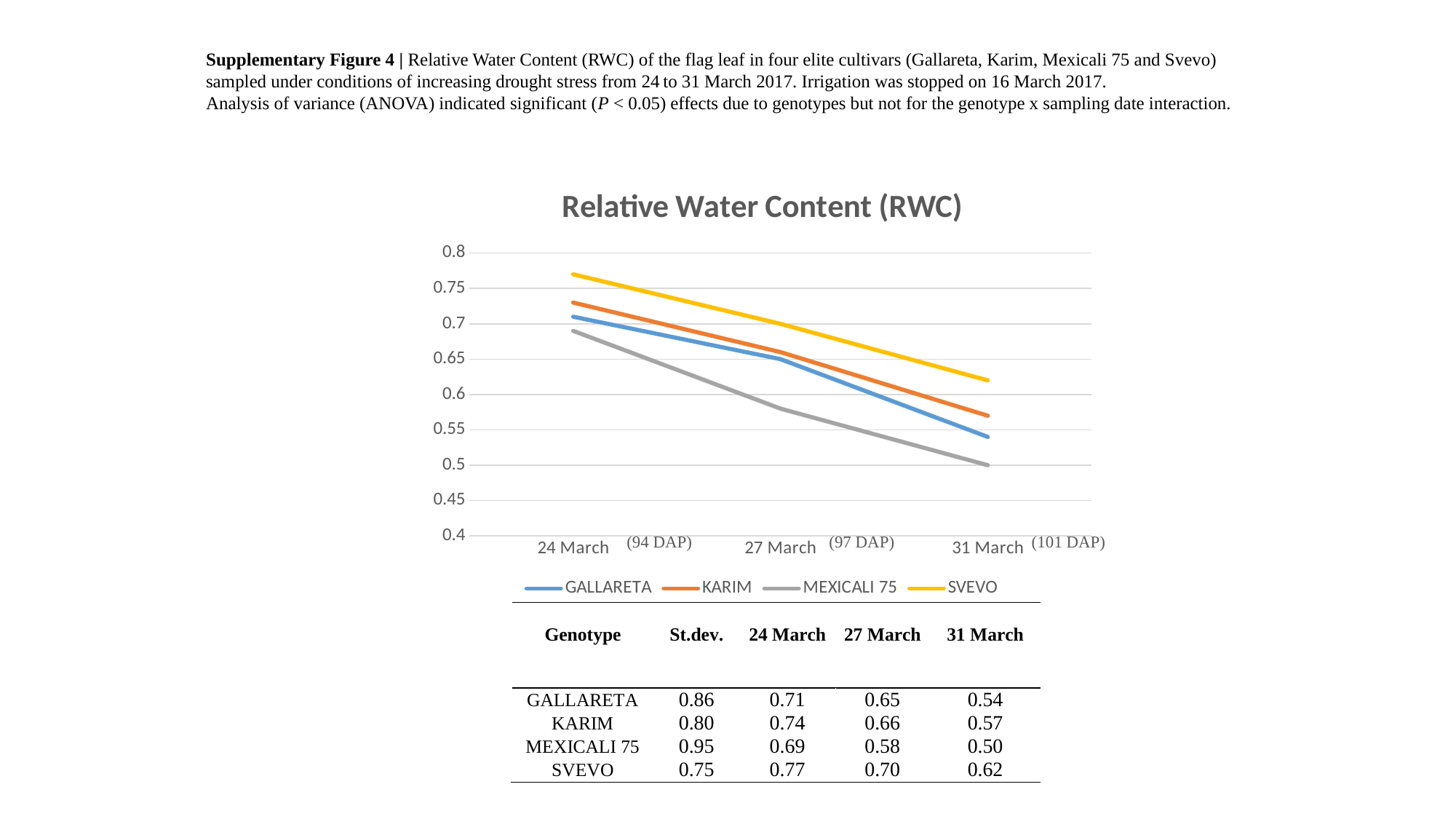

Supplementary Figure 4 | Relative Water Content (RWC) of the flag leaf in four elite cultivars (Gallareta, Karim, Mexicali 75 and Svevo)
sampled under conditions of increasing drought stress from 24 to 31 March 2017. Irrigation was stopped on 16 March 2017.
Analysis of variance (ANOVA) indicated significant (P < 0.05) effects due to genotypes but not for the genotype x sampling date interaction.
### Chart: Relative Water Content (RWC)
| Category | GALLARETA | KARIM | MEXICALI 75 | SVEVO |
|---|---|---|---|---|
| 24 March | 0.71 | 0.73 | 0.69 | 0.77 |
| 27 March | 0.65 | 0.66 | 0.58 | 0.7 |
| 31 March | 0.54 | 0.57 | 0.5 | 0.62 |(94 DAP)
(97 DAP)
(101 DAP)
