# Supplementary material for: Comparative Aerial and Ground Based High Throughput Phenotyping for the Genetic Dissection of NDVI as a Proxy for Drought Adaptive Traits in Durum Wheat
Source: Front Plant Sci. 2018 Jun 26;9:893. doi: 10.3389/fpls.2018.00893 (PMC6028805; doi:10.3389/fpls.2018.00893)
Supplement: Supplementary file 18 [file Presentation_5.PPTX]

## Slide 1
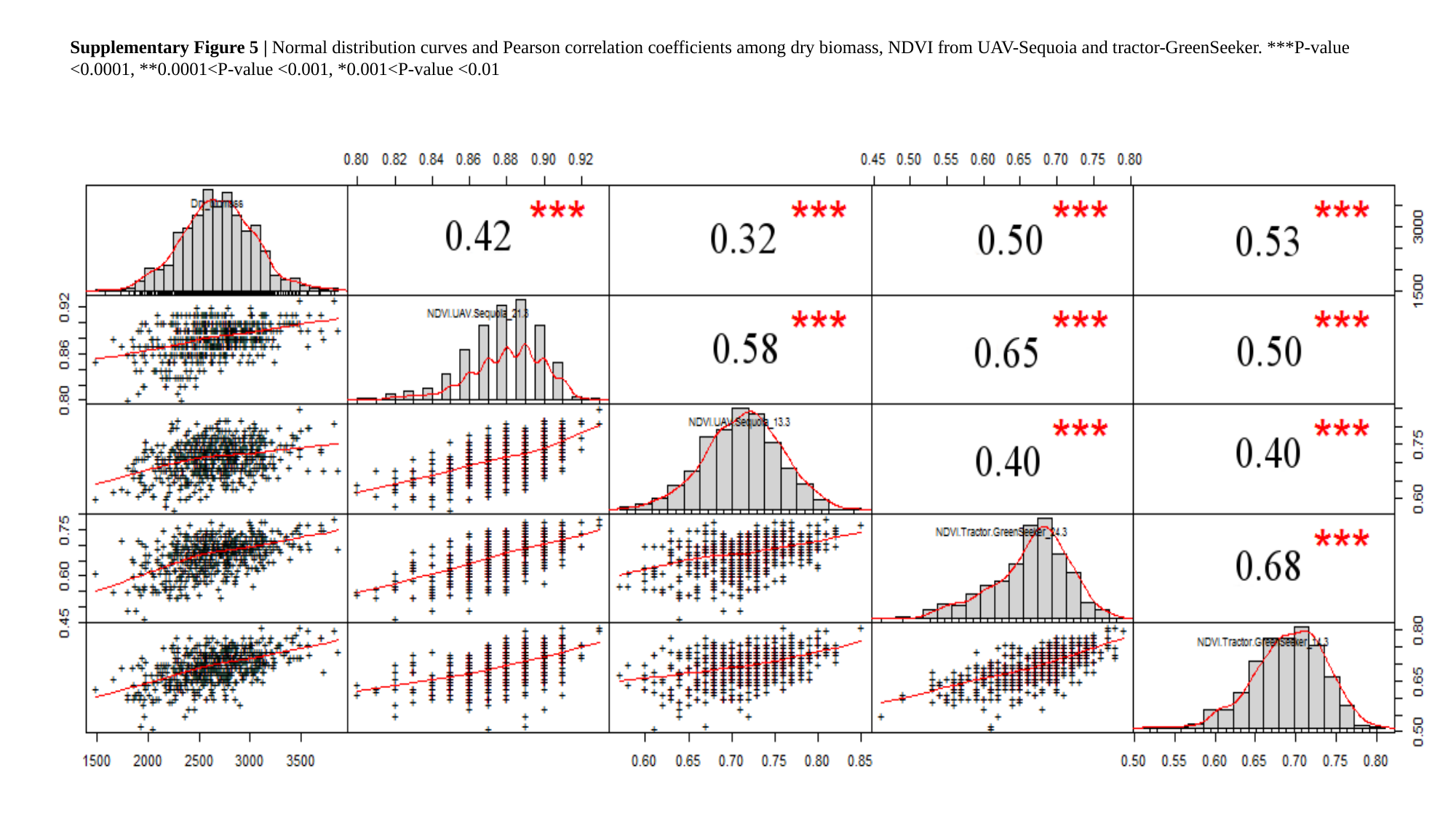

Supplementary Figure 5 | Normal distribution curves and Pearson correlation coefficients among dry biomass, NDVI from UAV-Sequoia and tractor-GreenSeeker. ***P-value <0.0001, **0.0001<P-value <0.001, *0.001<P-value <0.01
